# Recent Progress and Morphological Distribution of Polydopamine-Based Biomaterials and Their Applications

**DOI:** 10.3390/gels12030187

**Published:** 2026-02-24

**Authors:** Zoobia Bashir, Mahroza Kanwal Khan, Xueli Zhang

**Affiliations:** 1 College of Chemistry and Environmental Engineering, Shenzhen University, Shenzhen 518060, China; zoobiabashir@szu.edu.cn (Z.B.); mahrozakhan@gmail.com (M.K.K.); 2 Department of Biomedical Engineering, School of Medicine, Shenzhen University, Shenzhen 518060, China

**Keywords:** polydopamine, nanocomposite, nanoscale materials

## Abstract

Polydopamine (PDA) is a bioinspired polymer known for its strong adhesiveness, biocompatibility, and functional properties, making it highly useful in biomedical applications. This review highlights recent progress in PDA-based biomaterials, with a focus on their morphology, synthesis techniques, and various biomedical uses. It examines how PDA composites, which are formed at the nanoscale and macroscale levels, contribute to drug delivery, tissue engineering, wound healing, and cancer treatment. The ability of PDA to create stable, functional coatings and composites that bond well with different biomaterials enhances its therapeutic potential. This review also discusses challenges such as structural stability, toxicity, and production scale. Additionally, it covers different polymerization mechanisms and their implications for future clinical use. With ongoing advancements, PDA-based materials hold great promise for personalized medicine, including targeted drug delivery, photothermal therapy, and tissue regeneration. Overall, this overview emphasizes the vital role of PDA in the progression of biomedical technology and its potential for future applications.

## 1. Introduction

Composite materials have received much attention in recent years because of their better mechanical [1], electrical [2], thermal [3], and optical properties [4,5]. Polydopamine (PDA)-based composite materials are considered one of the most desirable materials in nanocomposites, as they are versatile and bioactive systems with tremendous potential in biomedical applications and other fields [6,7,8]. PDA is a polymer with adhesive properties that mimic the attachment abilities of mussels, demonstrating outstanding qualities such as exceptional biocompatibility, bioactivity, and strong adhesion to various surfaces [9]. These features make it a perfect candidate for various biomedical uses, such as drug delivery [10], tissue engineering [11], diagnostic imaging [12], and wound healing [13], among others. PDA is a type of polymer obtained from the process of the oxidative polymerization of dopamine (mildly basic conditions) [14]. This is a catecholamine-like polymer imitating adhesive proteins found in the adhesives of marine mussels [15]. PDA is a polymer that can be polymerized with rich surface chemistry containing hydroxyl, amine, and catechol functional groups that can react strongly with a wide range of materials, such as nanoparticles, metals, and biological molecules [16,17]. Owing to its bioactivity and adhesive nature, PDA is an essential material in the design of nanocomposites for biomedical applications [18]. The PDA polymer matrix and embedded nanomaterials [19], e.g., metallic nanoparticles [20], carbon nanomaterials [21], ceramics [22], or other working nanomaterials, are the two main components of PDA-based nanocomposites [23,24]. A specifically designed combination of properties in this unique composite makes it very useful in the biomedical field. Additionally, the morphology of PDA-based nanocomposites is a crucial factor in determining their biological and mechanical properties. Among the nanomaterials embedded in the PDA matrix, the area, shape, and size of the nanoconstructs can play essential roles in the behavior of these composites in medical applications [25,26].

The morphology and size of polydopamine (PDA)-based materials are important in their reliance on the biological and functional characteristics of the material. Smaller PDA nanoparticles (usually ranging between 1–100 nm) are useful for applications such as drug delivery because they offer better biodistribution, rapid cellular uptake, and a high rate of clearance by the kidneys [22]. Smaller PDA materials, including fibers or macromolecular coatings, on the other hand, are useful in tissue engineering and wound healing, where they offer superior mechanical strength, stability, and sustained release of therapeutic agents. Also, the morphology of PDA materials, including surface roughness and aspect ratio, can be influential on cell adhesion, proliferation, and immune response. As an example, rougher surfaces and mesoporosity can be used to improve cell-material interactions, which induce better tissue regeneration. This knowledge of these size and morphology thresholds is important as it determines how the material will perform in a biological system and its application to a particular biomedical use. This update seeks to bring out the practical implications of the various PDA sizes and shapes with distinct guidelines for designing and using them in diverse medical disciplines [20].

Several previous reviews have discussed PDA-based nanocomposites [27]. Nevertheless, no review has comprehensively examined the composition, morphology, and details of the factors influencing the application of these methods in the medical field. Moreover, we discuss the latest developments in PDA composites and their clinical applications. In this review, we present the history, chemistry, morphology, and applications of PDA composites, as well as their future prospects for clinical development.

We classify PDA biomaterials into nanoscale and macroscale levels on the basis of their dimensions. Nanoscale materials are those with dimensions in the range of hundreds of nanometers, typically 1–1000 nm. In contrast, macromolecular materials, such as coatings, sheets, and fibers, have at least one dimension in centimeters. The key morphological descriptors focused on include the particle size, aspect ratio, mesoporosity, shell thickness, and surface roughness, all of which significantly influence the material’s properties and applications. This classification helps provide a clearer understanding of the structural characteristics of PDA biomaterials.

The morphology of PDA biomaterials, including their particle size, aspect ratio, mesoporosity, shell thickness, and roughness, significantly impacts their biological performance. For example, surface roughness and mesoporosity enhance cell adhesion and proliferation by improving cell–material interactions, as observed in PDA-based hydrogels and composites [28]. Smaller particles in the nanoscale range tend to have better biodistribution and faster clearance, whereas larger macromolecular materials may accumulate in specific tissues and show slower clearance. Additionally, the size and shape of PDA materials influence immune activation. Smaller, irregularly shaped particles can be more readily recognized by the immune system, impacting their ability to induce immune responses or evade detection [29]. These relationships have been observed in previous studies, linking morphological features to enhanced biomedical applications.

The rate of polymerization of dopamine to PDA depends on the temperature and pH, with optimum pH ranging between 8 and 10, in which polymerization is maximized. Quantitative rate constants of the oxidation and polymerization of the PDA formation reaction, depending on past literature and experimental evidence. Also, the pH-dependence of the adhesion rates and drug release rate of PDA has been investigated, showing that PDA-based materials exhibit higher adhesion rates at neutral to slightly alkaline pH, which is very advantageous when applied in the context of tissue engineering. We have also conducted a mechanistic study of the interactions between PDA and metal ions or other substances, e.g., nanoparticles, via coordination bonds and π–π stacking interactions. This fine-grained, mechanistic quantification will enrich our knowledge about the behavior and performance of the material in different biomedical applications, which will be more firmly based on the subsequent research and clinical application.

**Figure 1:**
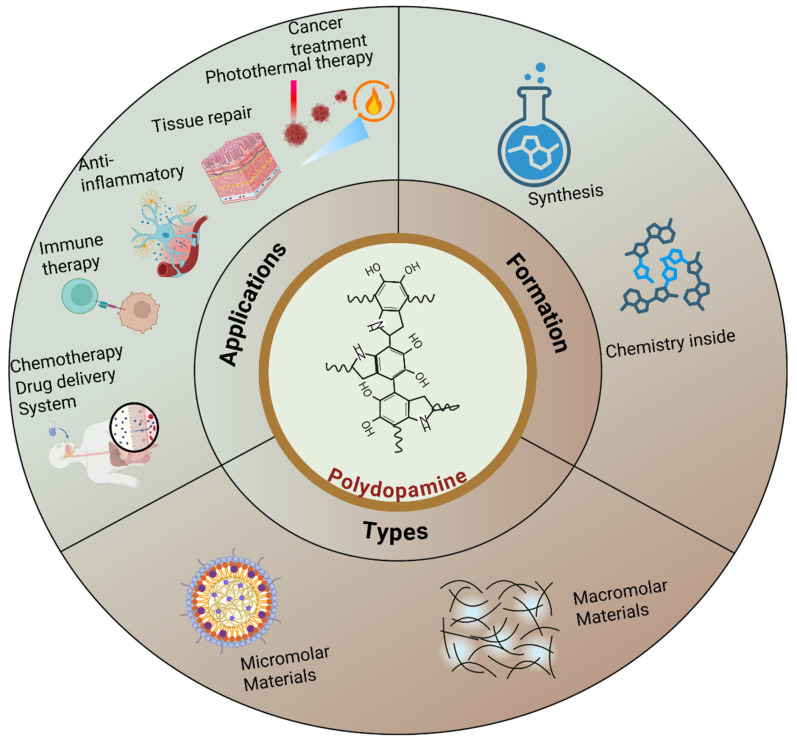
Graphical preview.

The degradation products of polydopamine (PDA) are primarily catechol derivatives and dopamine quinones, which are generally considered biocompatible. These products are broken down and eliminated from the body, with smaller nanoparticles typically cleared through the renal system via filtration by the kidneys. In contrast, larger macromolecular PDA particles tend to accumulate in organs such as the liver and spleen due to their size, leading to slower hepatic clearance. The morphology and size of PDA materials also influence their clearance, with irregularly shaped or larger particles being more likely to accumulate in the reticuloendothelial system (RES). This suggests that smaller particles, typically in the nanometer range, are more rapidly cleared via renal excretion, whereas larger particles have prolonged circulation times and may accumulate in hepatic tissues (Figure 1).

## 2. History

PDA is a synthetic polymer and, therefore, has attracted much attention as a result of its distinctive chemical features, properties, and ability to be used in numerous sectors, such as nanotechnology [30], materials science [31], and biomedical engineering [32]. A trace number of PDA-based nanocomposites have been used in the study of dopamine (l-3,4-dihydroxyphenylalanine), a naturally occurring molecule that is of key importance in biological processes [33]. The creation of PDA and its application in nanocomposites was inspired by the adhesion characteristics of mussels, which use a similar compound to attach to surfaces [34]. Dopamine (also known as 3,4-dihydroxyphenylethanol or DOPA) is a neurotransmitter whose stereochemistry is troublesome because of its catechol group, which renders it highly reactive toward chemicals [35,36]. In 2007, polymers of dopamine (PDA) were first characterized by Lee et al., who discovered that the self-polymerization of dopamine could be used to produce a stable, adhesive-like film under alkaline conditions. The discovery was path-breaking because it imitates the way mussels can naturally adhere to wet surfaces via the use of dopamine-based chemical substances to fix themselves [37]. This finding led to the emergence of PDA as a comprehensive biomimetic material, with its usage extending far beyond its initial intended use [38,39]. The ability of dopamine to self-polymerize at high pH results in PDA being able to be prepared under ambient conditions that do not require expensive reagents or sophisticated equipment. The PDA film formed during the polymerization of dopamine contains a myriad of functional groups that can bind with numerous surfaces and other materials, e.g., catechol and amine groups.

**Figure 2:**
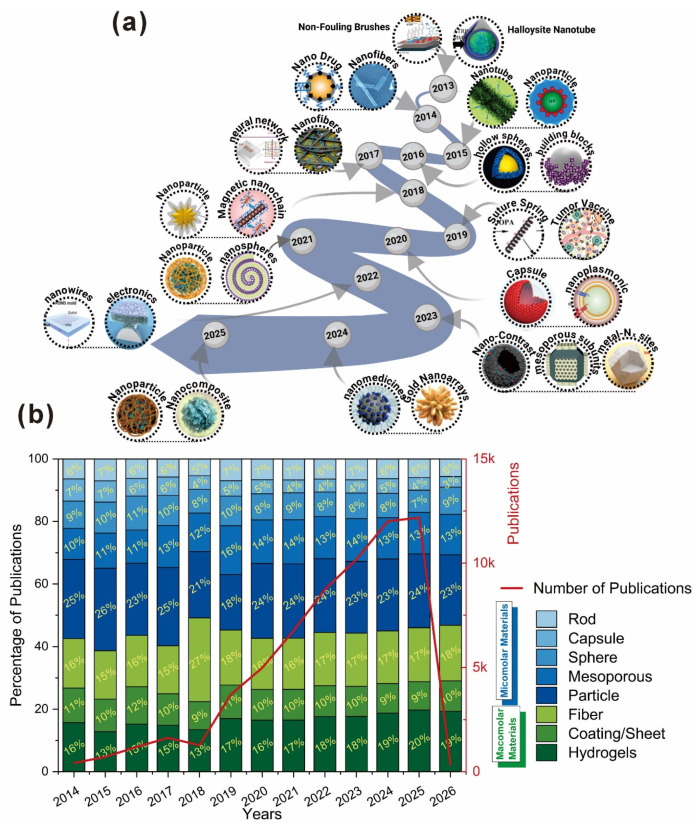
Histories of polydopamine-based composite materials. (**a**) Timeline of the research focus (2013–2025) [40,41,42,43,44,45,46,47,48,49,50,51,52,53,54,55,56,57,58,59,60,61,62,63,64,65,66] Copyright 2013, Journal of the American Chemical Society. Copyright 2013, Wiley. Copyright 2014, Journal of the American Chemical Society. Copyright 2014, Journal of the American Chemical Society. Copyright 2015, Journal of the American Chemical Society. Copyright 2015, Journal of the American Chemical Society. Copyright 2016, Nature Portfolio. Copyright 2016, Nature Portfolio. Copyright 2017, Nature Portfolio. Nature Portfolio. Copyright 2017, Nature Portfolio. Copyright 2018, Nature Portfolio. Copyright 2018, Nature Portfolio. Copyright 2019, Journal of the American Chemical Society. Copyright 2019, Journal of the American Chemical Society. Copyright 2020, Nature Portfolio. Copyright 2020, American Association for the Advancement of Science (AAAS). Copyright 2021, American Association for the Advancement of Science (AAAS). Copyright 2021, Nature Portfolio. Copyright 2022, American Association for the Advancement of Science (AAAS). Copyright 2022, American Association for the Advancement of Science (AAAS). Copyright 2023, Journal of the American Chemical Society. Copyright 2023, Nature Portfolio. Copyright 2023, Nature Portfolio. Copyright 2024, Journal of the American Chemical Society. Copyright 2024, Nature Portfolio. Copyright 2025, Journal of the American Chemical Society. Copyright 2025, Journal of the American Chemical Society. (**b**) Total number and percentage of publications published for each type of composite material from 2014 to 2026.

First, we search for the keyword “polydopamine biomedical field” in the “app dimension,” categorize the results by structural morphology, and download the relevant research articles. Figure 2 provides a comprehensive overview of the evolution and publication trends in PDA-based composite materials. Figure 2a shows how research topics and material types have developed over time, beginning with basic concepts such as nonfouling brushes and halloysite nanotubes in 2013 and then expanding to include complex structures such as nanowires, nanomedicine, and gold nanospheres by 2025. This trend indicates a gradual move toward more specialized applications, including drug delivery, tumor vaccines, and nanoscale composites. Figure 2b highlights the increasing number of publications from 2014 to 2026, with a sharp rise after 2021, indicating rapid growth in the field. The bar chart also shows the distribution of materials within the PDA composites, with nanoparticles and mesoporous structures being the most common, followed by hydrogels and capsules. These insights, obtained through the Appdimension tool, reflect not only the accelerating rate of innovation but also the expanding variety of material applications in PDA-based composites, demonstrating a shift toward more complex and functional designs in recent years.

## 3. Chemistry Inside PDA Biomaterials

**Figure 3:**
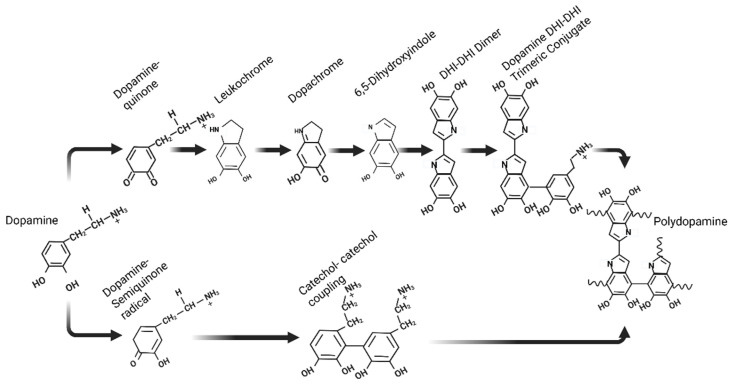
Polymerization and interaction mechanisms of dopamine. PDA formation: a copolymer composed of 5,6-dihydroxyindole (DHI) and dopamine.

No material-dependent surface chemistry process has been developed that alters the properties of virtually any material surface more than does PDA coating. There are several mechanisms through which catechol can interact with surfaces, and these mechanisms are dependent on the type of surface [67]. These mechanisms include metal coordination, Michael-type additions and/or Schiff-base formations, hydrogen bonding, and π–π stacking, among others [68]. Despite extensive studies conducted over the last decade, understanding the detailed mechanism underlying the formation of polydopamine (and its related catecholamine derivatives) remains a challenging endeavor. The structure of PDA is not easy to understand because of the heterogeneity within a monomeric unit. The following sections provide an overview of some of the monomer structures incorporated into PDA. First, 3,4-dihydroxyindole (DHI), which is spontaneously generated from dopamine (the upper pathway), is widely accepted as a predominant building block of enzymes [67]. By degrading PDA, pyrrole derivatives are produced by hydrogen peroxide in the presence of pyrrole-2,3-dicarboxylic acid and pyrrole-2,3,5-tricarboxylic acid [69]. This is direct evidence of the presence of DHI in PDA. A second notable feature is the presence of uncyclized oxidized dopamine, which contains primary amine groups on its surface. A catechol-to-catechol reaction subsequently reacts with either DHI or dopamine through the formation of dopamine–quinone [70]. A single electron is lost by external stimuli such as light irradiation, resulting in the formation of a dopamine-semiquinone ring (Figure 3). A dopamine-semiquinone radical is either further oxidized intramolecularly into dopamine-quinone, followed by DHI formation (Figure 3), or intermolecularly oxidized to cause dimerization, yielding a catechol-catechol dimer [71].

### 3.1. Dopamine and Catechol Derivatives

Biochemistry and material science rely on dopamine and catechol derivatives to function. Dopamine is a neurotransmitter and a precursor to many chemical compounds in the brain. Catechols, for example, are phenolic compounds with hydroxyl groups at both the 3 and 4 positions of the benzene ring. Surface chemistry extensively uses PDA coatings to modify surface properties [72].

**Figure 4:**
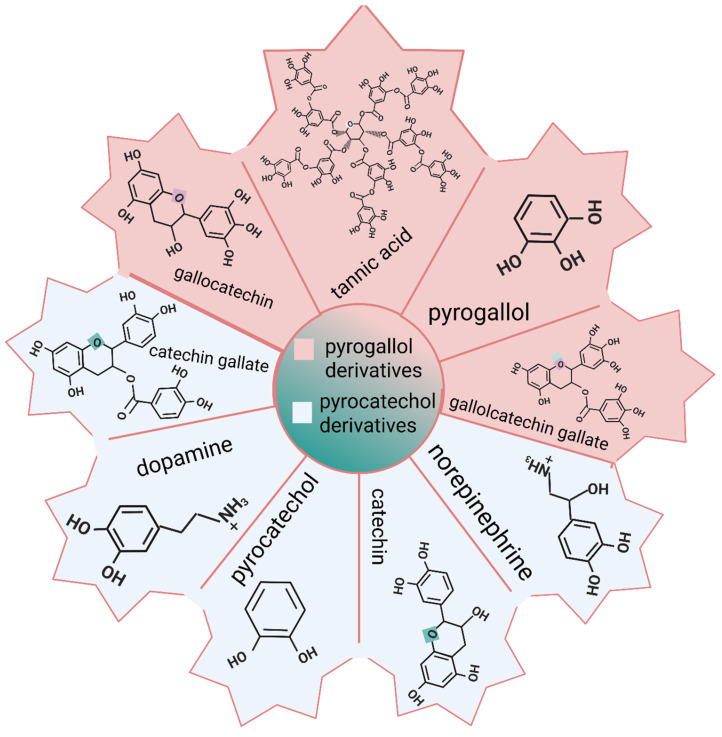
Diverse catechol or catecholamine derivatives. Class A: Catechol derivative small molecules (blue): pyrocatechol, catechin, dopamine, norepinephrine, and catechin gallate; Class B: gallol derivative small molecules (pink): pyrogallol, gallocatechin, tannic acid (TA).

Instead of focusing on the antioxidant properties of polyphenols, their study revealed that they also function as underwater adhesives. A variety of synthetic phenol-containing polymers have been reported to demonstrate waterproof or wet-resistant adhesive properties; however, none of these materials can demonstrate adhesive properties on a wide variety of materials, as is the case with marine mussels in nature. Phenol and amine moieties in mussel adhesive proteins bind to virtually any surface under water, similar to the natural ability of mussels. In the field of surface functionalization, PDA coating is the first material-independent method of surface modification. In addition, molecules such as norepinephrine, poly(ethylenimine)-catechol, or chitosan-catechol, which contain both phenol (especially catechols) and amine groups, exhibit material-independent surface coatings similar to those of PDA. Molecular weight and configuration can be used to categorize studies into four categories [73]. As shown in Figure 4, catechol-containing small molecules, gallol-containing small molecules, catechol-tethered polymers, and/or gallol-tethered polymers can be distinguished.

### 3.2. Polymerization and Surface Functionalization

Chemistry of polyphenols, focusing on small molecules such as dopamine, norepinephrine, tannic acid, catechins, and related compounds. The surface can be functionalized through the oxidative polymerization of the molecules themselves without the need for conjugation to polymers in aqueous buffers and solvents. Instead of discussing the catechol and gallol groups in the previous section, we discuss the covalent conjugation of these groups to polysaccharides for the preparation of hydrogels and other materials. As a first step, catechol-conjugated cationic polysaccharides such as chitosan-catechol [74,75,76,77] and glycol-chitosan-catechol [78], as well as anionic polysaccharides such as alginate-catechol [79,80,81] and hyaluronic acid-catechol [81,82,83], are presented. Furthermore, a new class of gallol-conjugated polysaccharides is described.

**Figure 5:**
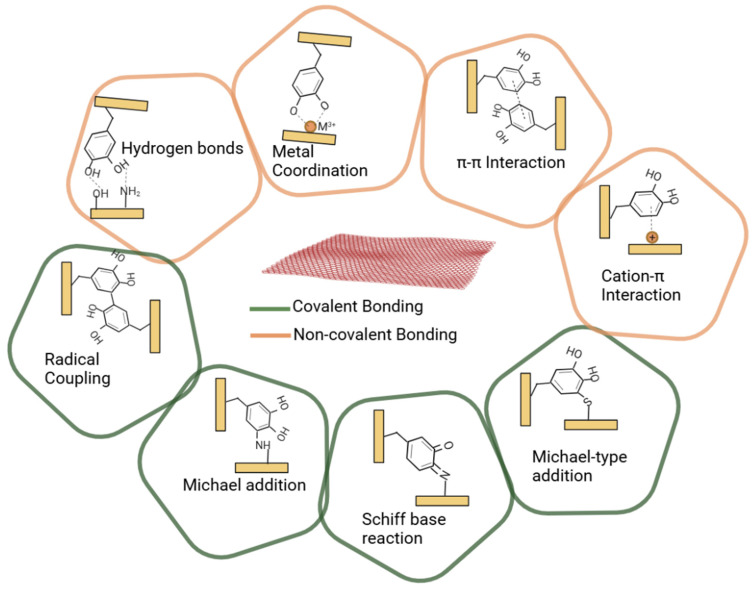
Surface reactivity mechanisms of catechol-conjugated polymers for hydrogel formation. Green circle: Covalent crosslinking; orange circle: Noncovalent crosslinking.

Importantly, all the polymer chains with phenol-conjugated groups undergo reversible physical interactions such as π–π interactions, π–cation interactions, hydrogen bonding, and metal coordination or irreversible covalent bonds with adjacent functional groups (e.g., catechol/gallol and amine groups) (Figure 5). During covalent bond formation, catechol groups spontaneously oxidize to form o-catecholquinone, an electrophilic intermediate. During this reaction, catechol-to-catechol adducts and catechol-to-amine (or thiol) adducts form. Additionally, the aforementioned catechol adduct can form at the tissue interface, resulting in a strong bond between the two tissues.

Polydopamine (PDA)-based materials have particular strengths and drawbacks compared with other materials, including PLGA (poly(lactic-co-glycolic acid)) and rMAP (recombinant mussel adhesive proteins), and depend on the intended use. PDA is very strong in its adhesive forces, biocompatibility, and ease of functionalization; hence, its use in drug delivery, wound healing, and tissue engineering is ideal. Compared to PLGA, which is mostly employed in controlled drug delivery because of its biodegradability, PDA has a high adhesion to biological surfaces, making it useful in processes that demand stable covers or tissue attachment. Moreover, PDA-based materials can be modified with nanoparticles, metals, and other biomolecules, whereas, compared to PLGA, PDA-based materials are more flexible in terms of functionalization. In contrast, rMAPs, which also provide good adhesion capabilities, are generally more application-specific to direct tissue adhesion and regeneration, and might not have the same general chemical capabilities as PDA does. Although PDA is quite efficient in most instances, PLGA can be better applicable in areas where biodegradability and low degradation rates are paramount, and rMAPs can be more applicable to particular regenerative applications. This comparison highlights the unmatched advantages of materials based on PDA, especially in the multifunctional and high biointerface interactions applications.

In a bid to solve the technical bottlenecks, production capacity, and cost estimates of polydopamine (PDA)-based materials, we have discovered that there are major challenges and opportunities. The polymerization process is the most crucial step in the large-scale production of PDA since it is slow and complicated, and therefore, it may be a limiting factor in scalability. Nevertheless, the optimization of the synthesis parameters, including dopamine concentration, temperature, and the reaction time, might be useful to simplify production and enhance the efficiency. On the issue of production capacity, we project that with the existing techniques, the production scale could be up to 1000 kg (or more) per year with large-scale production based on the current techniques, but more process optimization is required to satisfy the needs of the industry. On a cost basis, the key factors are the cost of raw materials (e.g., dopamine), the reaction time, and special equipment required in the process of polymerization. The early cost analysis indicates that to produce PDA-based materials on a large scale would be costly by about a range of c. $3–12/g in cost, but the process optimization and economies of scale may reduce the costs. These lessons indicate that PDA-based material production can be scaled up, but more research and development are needed to lower the cost of production and address the technical issues.

## 4. Structural Distribution

**Figure 6:**
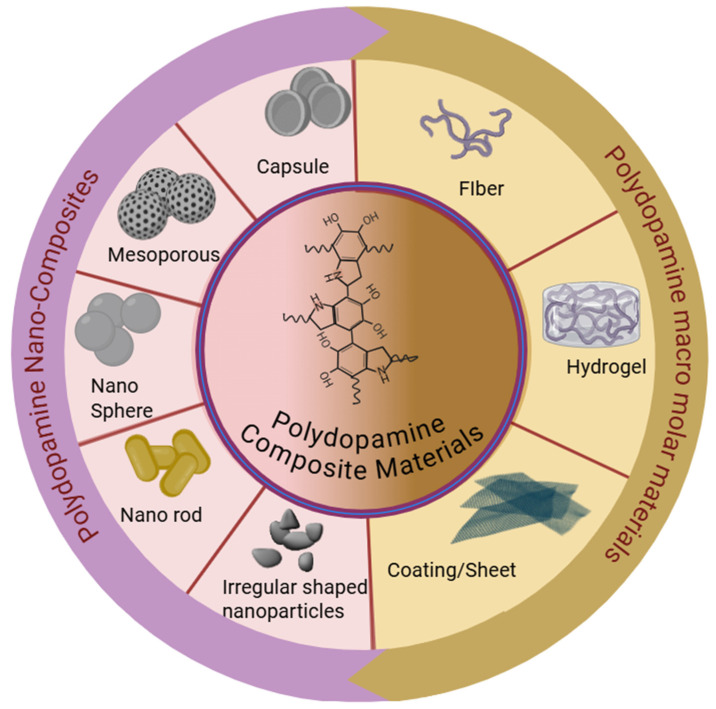
Schematic figure of the types of PDA nanocomposites.

The structural dispersion of PDA-based composites is a crucial factor in determining their performance in biomedical applications. The combination of these materials with the PDA matrix leads to unique properties of these composites, such as the use of metal nanoparticles [84,85,86], carbon-based materials [87,88], or ceramics [89]. The PDA coating typically creates a consistent film around the nanoparticles, thereby providing a stable interface between the nanoparticles and their environment [90,91,92,93]. The PDA surface has catechol and amine functionalities that promote robust interactions with the nanoparticles [20,94,95], and this provides favorable dispersity as well as homogeneity of the distribution in the composite [5,96]. By varying the synthesis parameters, such as the dopamine concentration, nanomaterial type, and polymerization time, the structural distribution of the nanoparticles within the PDA matrix can be adjusted (Figure 6). This will enable the manufacturing of composites with a predictable morphology, i.e., hydrogels, sheets, and fibers (macromolecular materials) (Table 1), as well as nanoparticles, nanospheres, mesopores, and nanorods, among other nanomaterials. The surface area also increases due to the even dispersion of nanoparticles, a critical factor for the application of biomaterials in various clinical settings (Table 2).

### 4.1. Polydopamine-Based Hydrogels

PDA-based hydrogels are biomaterials that mimic the adhesive properties of natural mussel adhesive proteins. They are formed by polymerizing dopamine, resulting in a network with excellent mechanical properties and the ability to absorb water. Owing to their versatility and bioactive surface, these hydrogels are used in drug delivery [97], tissue engineering [11,98,99], and wound healing [100,101] applications.

**Figure 7:**
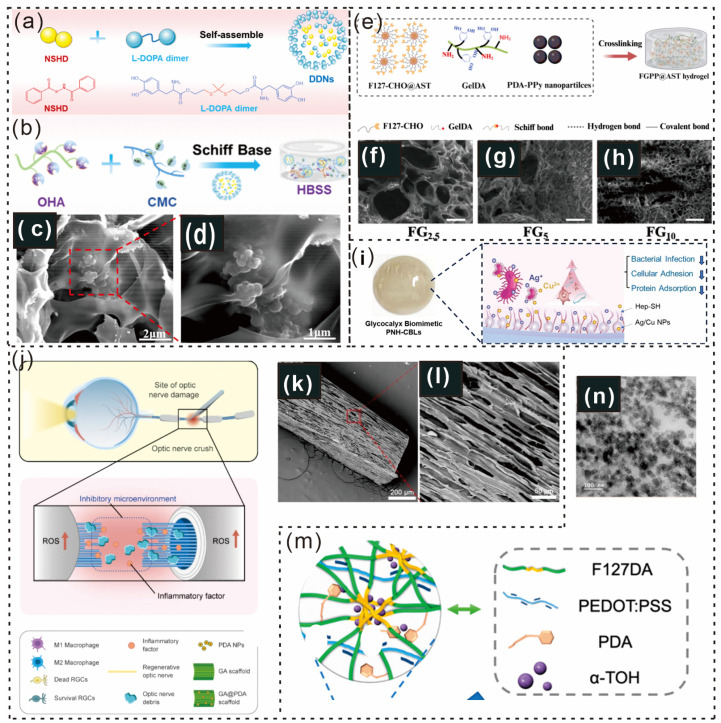
Schematic illustration and microscopic pictures of PDA-based hydrogels (**a**) showing the synthetic process of DDNs via the self-assembly of NSHD and L-DOPA dimers. (**b**) Integration of DDNs into the hydrogel network through spontaneous Schiff base-ligation, enabling hydrogel formation. (**c**,**d**) Scanning electron microscopy (SEM) images of the HBSS samples [102]. Copyright 2025, American Chemical Society. (**e**) Schematic representation of the preparation process for the multifunctional F127CHO-GelDA-PDA-PPy@AST (FGPP@AST) hydrogel. (**f**–**h**) SEM images demonstrating the pore structures of hydrogels with varying GelDA concentrations (FG2.5, FG5, and FG10) (scale bars: 500 μm) [103] Copyright 2025, Elsevier. (**i**) The structure and function of the glycocalyx in ocular health [104]. Copyright 2024, Elsevier. (**j**) The therapeutic mechanism of ROS-scavenging biomimetic scaffolds (GA@PDA) in protecting retinal ganglion cells (RGCs) and promoting axonal regeneration by modulating the inhibitory microenvironment following optic nerve injury. (**k**,**l**) SEM images of longitudinal sections of the GA@PDA scaffold [107]. Copyright 2024, SpringerLink. (**m**) F127DA-PEDOT/PSS-PDA-α-TOH (FPDA) hydrogel structure. (**n**) TEM images of a 10% *w*/*v* F127DA hydrogel solution loaded with 10 mg/mL α-TOH (scale bar: 100 nm) [108] Copyright 2024, American Chemical Society.

A multifunctional HBSS for dressing skin burn wounds (SBWs) was designed to accelerate wound regeneration through localized H_2_S delivery while preventing postinjury bacterial infections (Figure 7a,b) [102]. Dopamine dimers that form nanoscale assemblies (DDNs) were successfully incorporated into the aldehyde-modified hyaluronic acid and carboxymethyl chitosan (OHA-CMC) hydrogel matrix, with a nanoparticle loading of approximately 85%, as shown by environmental scanning electron microscopy (ESEM) (Figure 7c,d) [102]. Furthermore, to address complex therapeutic challenges, Xiaopei Li and colleagues developed a biodegradable, multifunctional hydrogel patch that offers myocardial adhesion, mechanical reinforcement, enhanced electrical conductivity, and targeted drug release within the infarct microenvironment (Figure 7e) [103]. Scanning electron microscopy (SEM) revealed variations in the pore structure and network density corresponding to different GelDA concentrations in the FG hydrogels (Figure 7f–h) [103].

Dai et al. reported that the ocular surface glycocalyx is a critical barrier between the ocular epithelium and the external environment [104]. Extending 500 nm from the plasma membrane plays key roles in preventing microbial invasion, maintaining tear stability, preserving antibacterial biomolecules, and regulating ocular surface homeostasis (Figure 7i) [104]. Additionally, a biopolymer-based scaffold (GA@PDA) with an optic nerve-mimicking microstructure was fabricated via the ice-templating method. Antioxidative PDA nanoparticles (NPs) have been incorporated as ROS scavengers to modulate microglia/macrophage polarization [105,106]. The scaffold’s biocompatibility and antioxidative and anti-inflammatory properties were assessed both in vitro and in vivo [107]. The nanocomposite scaffold reduced oxidative stress, thereby promoting the polarization of microglia/macrophages from the M1 phenotype to the M2 phenotype (Figure 7j) [107]. SEM analysis revealed that PDA NP incorporation did not significantly affect the microstructure of the scaffold (Figure 7k,l) [107].

PDA hydrogels have significant potential across various biomedical fields, including drug delivery, tissue engineering, and wound healing, owing to their unique adhesive properties and bioactive surfaces. Recent advancements, such as the development of multifunctional hydrogels for burn wound treatment and myocardial repair, underscore their versatility and effectiveness in addressing complex therapeutic challenges.

In another study, Zhang et al. utilized diacrylated Pluronic F127 micelles as macrocross-linkers for a hydrogel loaded with the hydrophobic drug α-tocopherol (α-TOH) [109]. By in situ synthesis of PDA and the integration of conductive components, an injectable and highly flexible antioxidant/conductive composite FPDA hydrogel was developed (Figure 7m). TEM images confirmed the well-dispersed nature of α-tocopherol (α-TOH) in solution (Figure 7n) [108]. Furthermore, the compositions and applications of the PDA-based hydrogels are presented in Table 1.

While hydrogel-based systems offer promising advantages such as biocompatibility and self-healing properties, several key limitations remain. First, their mechanical strength and stability under dynamic conditions, especially in environments such as the heart or skin, need significant improvement, as many formulations suffer from rapid degradation and insufficient durability [102]. Second, the swelling and degradation behaviors of hydrogels depend heavily on the crosslinking density, which, if too high, can hinder nutrient diffusion and cell viability. Finally, although hydrogels are effective for drug delivery, challenges persist in achieving controlled and sustained release, particularly for hydrophobic drugs, which impacts the overall therapeutic efficacy [103]. Addressing these gaps is crucial for optimizing hydrogel performance in clinical applications.

### 4.2. Polydopamine Coatings/Sheets

In recent years, PDA coatings have gained importance because of their distinctive properties, such as strong adhesion and high versatility. PDA coatings are described here in the context of their synthesis. There is a particular focus on their versatility and potential research directions in this area, as well as the benefits and challenges connected with them.

**Figure 8:**
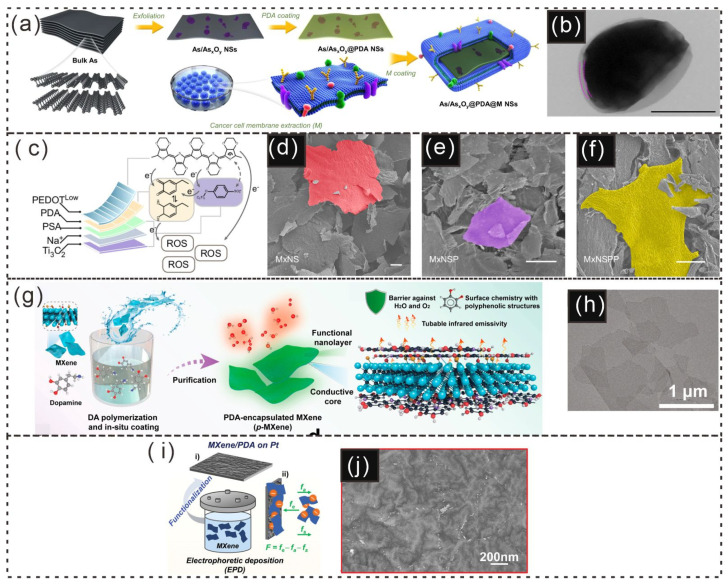
Schematic illustration and microscopic pictures of PDA-based coatings. (**a**) Schematic illustration of the preparation of As/As_x_O_y_@PDA@M NSs. (**b**) TEM images of as/As_x_O_y_@PDA@M NSs; scale bar = 100 nm [28]. Copyright 2021, Nature Portfolio. (**c**) Illustrations of the MxNSPP nanosheet composite and its ROS-responsive properties. (**d**–**f**) SEM images of sulfonic MXene nanosheets (MxNS), PDA-functionalized sulfonic MXene nanosheets (MxNSP), and MxNSPP (scale bar: 20 µm) [29] Copyright 2023, United States National Academy of Sciences. (**g**) Schematic representation of the preparation process for p-MXene. (**h**) TEM image of p-MXene nanosheets [111]. Copyright 2022, American Chemical Society. (**i**) Schematic illustration of the fast electrodeposition process for MXene/PDA composite electrodes, with a diagram of MXene electrophoretic migration shown in (i,ii). (**j**) SEM images of MXene/PDA-coated electrodes [112]. Copyright 2024, Wiley-VCH.

The synthesis of 2D ultrathin As/As_x_O_y_@PDA@M nanosheets (NSs) is shown in Figure 8a, where ball-grinding and liquid exfoliation techniques were employed, followed by PDA and cancer cell membrane coating. The successful deposition of the cell membrane coating on the surface of the As/As_x_O_y_@PDA NSs was confirmed by TEM, which revealed an obvious coating layer (Figure 8b) [28]. Baoning Sha synthesized ROS-responsive PDA-PEDOT-functionalized sulfonic MXene nanosheets (MxNSPP) via surface modification and self-assembly in solution. Additionally, nanosheets without a PEDOT layer (MxNSP) and those lacking both PEDOT and PDA layers (MxNS) were synthesized to investigate the impact of different layers on self-assembly and the immune response. Scanning electron microscopy (SEM) images (Figure 8c–f) revealed differences in morphology and thickness among MxNS, MxNSP, and MxNSPP [29].

Furthermore, strong electrostatic interactions between MXenes and dopamine (DA) can lead to flocculation during surface modification with PDA [9,110]. Deng et al. used a prepolymerization step: dopamine was first prepolymerized for 2 h in a Tris-buffer solution (pH ≈ 10) under agitation [111]. The resulting reactive PDA prepolymer was then used to decorate the MXene surface (p-MXene), forming covalent and hydrogen bonds and creating a uniform protective nanolayer that minimized sedimentation (Figure 8g). The nanolayer structure of p-MXene was similar to that of MXene (Figure 8h) [111].

Moreover, Qi Zeng and colleagues studied the formation of MXene/PDA composites via electrophoretic deposition (EPD), and a schematic diagram of electrophoretic migration under an electric field is shown in Figure 8i [112]. The corresponding SEM image is shown in Figure 8j [112]. PDA sheets/layers exhibit exceptional versatility and functionality, making them suitable for a wide range of applications, as discussed in Table 1. PDA coatings exhibit remarkable versatility, enabling a wide range of applications from drug delivery to surface modification in nanosheet synthesis. Despite challenges in maintaining uniformity and stability during synthesis, ongoing advancements in surface functionalization and self-assembly techniques are unlocking new potential in areas such as cancer therapy, bioelectronics, and biomaterial development.

Despite promising advances in polydopamine (PDA) coatings, several limitations remain to be addressed. First, the long polymerization times required for PDA deposition can hinder scalability and efficiency, especially in large-scale applications. Accelerating or optimizing the polymerization process for faster industrial implementation remains a significant challenge [29]. Second, while PDA coatings enhance adhesion and exhibit antioxidant properties, their long-term stability under dynamic environmental conditions (e.g., moisture and temperature fluctuations) remains to be investigated to ensure durability across various applications [111]. Finally, the interaction between PDA and different substrate materials can be complex, and more research is needed to understand how to tailor PDA modifications precisely to optimize functional properties, such as electrical conductivity and mechanical strength, especially in flexible bioelectronic devices [112].

### 4.3. Polydopamine Fibers

PDA fibers are synthetic structures created by polymerizing dopamine onto fiber substrates, mimicking the adhesive properties of natural materials. These fibers exhibit excellent mechanical strength and the ability interact various bioactive molecules. They are utilized in multiple applications. The types of formations and their uses in the clinic are discussed in Table 1.

**Figure 9:**
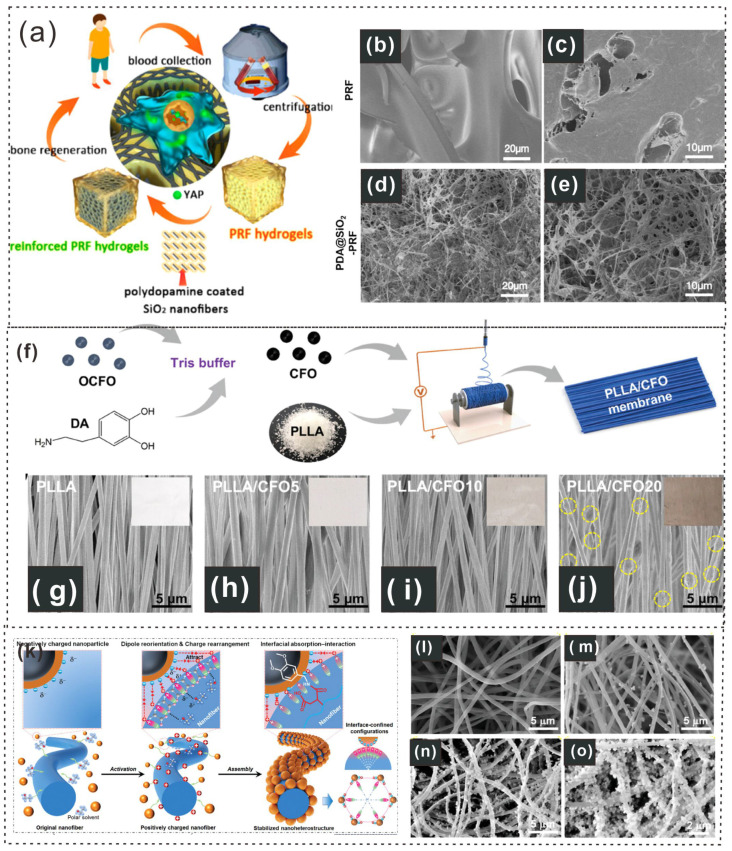
Schematic illustration and microscopic pictures of PDA-based fibers. (**a**) Schematic representation of the design and construction of PDA@SiO_2_-PRF for bone regeneration. (**b**–**e**) Digital photographs showing the injectability and moldability of the PDA@SiO_2_-PRF hydrogels during the sol–gel transition [114]. Copyright 2022, American Chemical Society. (**f**) Preparation of PLLA/CFO membranes through surface modification of PDA onto CFO and aligned electrospinning technology. (**g**–**j**) SEM images of the PLLA/CFO membranes, with insets showing digital photos [116]. Copyright 2024, Wiley-VCH. (**k**) Bioinspired multistimulus-responsive piezoelectric polymeric nanoheterostructures. (**l**–**o**) SEM images of PNHs fabricated with different concentrations of nanoparticle suspensions: (**l**) 0 wt% (pristine nanofibers), (**m**) 0.01 wt%, (**n**) 0.5 wt%, and (**o**) 1.0 wt% [117] Copyright 2024, Wiley-VCH.

The reinforced structure of the hybrid hydrogels provides bone ECM-like functions to stimulate osteoblast differentiation via the YAP signaling pathway [113]. From the biochemical perspective of Ren et al., the PDA components in blood-derived protein hydrogels (PDA@SiO_2_-PRF) slow the degradation of PRF, thereby ensuring the sustained release of autologous growth factors and preventing initial burst release [114]. This results in sustained osteogenic capacity, significantly promoting osteoblast differentiation and bone regeneration both in vitro and in vivo (Figure 9a). SEM images revealed that the PDA@SiO_2_ component was uniformly dispersed within the fibrin network of the PRF, interweaving with each other (Figure 9b–e) [114].

Additionally, Qian Zhang and colleagues showed that CFO agglomeration can reduce magnetic responsiveness, whereas inadequate interfacial compatibility hampers magnetomechanical transfer [115]. PDA has been widely used to improve the interfacial bonding between nanoparticles and the polymer matrix [115]. Therefore, CFO nanoparticles were decorated with PDA before hybrid electrospinning with PLLA (Figure 9f) [116]. By adjusting the CFO loading, a series of PLLA/CFO magnetoelectric nanofibrous membranes was fabricated. The color of the membranes shifted from white to brown as the CFO loading increased from 0 to 20 wt% (Figure 9g–j).

Furthermore, polymeric nanoheterostructures (PNHs) exhibit multifunctionality, converting various energy sources into electricity and enabling self-powered multistimulus detection, distinguishing them from conventional piezoelectric polymer materials [118,119]. Defeng Cui and colleagues reported that this innovative fabrication method employs a mild but effective nanostructure interface engineering strategy to assemble two distinct piezoelectric nanostructures into a stable, hierarchical nanoheterostructure (Figure 9k) [117]. The successful formation of PNHs with tunable morphologies was confirmed via SEM, with morphological changes depending on the nanoparticle suspension concentration (Figure 9l–o) [117]. PDA fibers exhibit outstanding adhesion and functionalization properties, making them suitable for diverse biomedical applications (Table 1). PDA fibers, owing to their excellent mechanical properties and ability to functionalize with bioactive molecules, offer great promise for a variety of biomedical applications, including skin tissue regeneration, wound healing, and bone regeneration. The versatility of these materials in enhancing interfaces, improving magnetic responsiveness, and supporting drug release underscores their potential in advancing therapeutic strategies and bioengineering innovations.

Polydopamine fibers, while versatile for various biomedical applications, face several key limitations. The scalability of their production remains a challenge because of the time-consuming oxidative polymerization process, which can be difficult to replicate at larger scales without compromising quality [116]. Additionally, these fibers often exhibit weak mechanical properties, limiting their use in load-bearing applications or structural scaffolds. Furthermore, although polydopamine is generally biocompatible, the long-term effects of its degradation products in the body remain underexplored, warranting further in vivo studies [117]. Addressing these gaps is essential for advancing the clinical use of polydopamine-based materials.

**Table 1:** PDA macromaterials, their applications, and other components used in them.

Type of Macromolar Martial	Name	Use	PDA Functional Requirement	Advantages	Disadvantages	Other Functional Components
**Hydrogels**	GelMA-MPF (GMPF)	Skin wound healing, tissue regeneration	Enhances adhesion, self-assembly, and antifibrosis	Antibacterial properties, Biocompatibility	Limited structural stability	Fibrinogen, copper ions [120]
	N.D.	Wound healing, tissue regeneration	Facilitates bioactive compound delivery via self-healing	Strong adhesion properties	Limited spatiotemporal control	Dopamine-modified cellulose, chitosan, Vitamin C, Mangiferin [121]
	UPA microspheres	Gout treatment	Polymerizes dopamine, enhances drug release	Targeted, controlled release	N.D.	Uricase [122]
	PDA-modified hydrogel	Periodontal bone healing, tissue regeneration	Antioxidative, immunomodulatory, conductive	Promotes mesenchymal stem cell (MSC) migration, angiogenesis	Limited by diabetes-induced inflammation	Poly(3,4-ethylenedioxythiopene)-assembled silk microfiber (PEDOT-PSF) [123]
	Lv/Hb-PDA-based Supramolecular Gel	Cancer Therapy, Tumor Microenvironment Targeting	Facilitating Gelation, Tumor Oxygenation	Enhanced Ferroptosis, Immunogenic Cell Death	Gel Degradation Over Time	Lovastatin, Hemoglobin, Catechol Groups [124]
	HPC/GPC/PFD	Diabetic foot ulcer healing	Stimulates controlled release of pirfenidone	Antibacterial effects, Biocompatibility	Cytotoxicity concerns	Reduced graphene oxide, fullerene, hyaluronic acid [125]
	Pul-SH/PDA/MoS_2_	Electronic skin, wearable technology	Improves adhesion to wet tissue and flexibility	Enhances tissue adhesion	Potential network defects	Graphene oxide, pullulan, molybdenum disulfide [126]
	NGF-AGHC	Nerve repair	Guides axons with conductivity and topographical cues	Cell adhesion promotion	Limited mechanical strength	Reduced graphene oxide, poly(vinyl alcohol), PDA [127]
	N.D.	Cartilage regeneration	Enhances cell differentiation via the piezoelectric effect	Enhanced stability	Limited cellular internalization	Barium titanate, graphene oxide [128]
	PCBUT (Polyzwitterionic hydrogel)	Infected diabetic wound healing	Regulates wound microenvironment	Antioxidant capacity	Complex preparation process	Carboxybetaine urethane acrylate, zwitterionic monomer [129]
	PNI-PAAM/PDA Hybrid Nanogels	Cancer immunotherapy	Captures and delivers antigens to dendritic cells	Strong adhesive properties, High stability	Cytotoxicity at high doses	Manganese dioxide, magnetic metal–organic framework [130]
	HD/alum/ICG hydrogel	Immunophototherapy for cancer	Provides CD8+ T-cell immune responses	Photothermal properties	Limited mechanical properties	Alum, ICG [131]
	CLDAFR hydrogel	Chronic pain-exacerbated myocardial reperfusion injury	Targets SCG, controlled drug release	Enhanced tissue compatibility	Limited degradation rate	Celecoxib, ropivacaine [132]
	C60@PDA/GelMA hydrogel	Skin wound healing	Scavenges ROS, promotes tissue regeneration	Tissue adhesiveness, Antibacterial capacity	Thermal instability	Fullerene nanocomposites, GelMA [133]
	FPDA hydrogel	Cardiac repair post-MI	Promotes antioxidant/conductive properties	N.D.	N.D.	α-Tocopherol, Pluronic F127 [108]
	HBSS hydrogel	Burn wound healing	Stimulates regenerative gas signaling, eliminates pathogens	Antibacterial properties, Promotes healing	Cytotoxicity at high doses	N-(benzoyl mercapto) benzamide [102]
	PNH-CBLs	Bacterial keratitis treatment	Provides antibacterial effects	Antibacterial, Surface modification	Cytotoxicity, Slow deposition	Ag/Cu bimetallic nanoparticles, Heparin [104]
	Alum-Tuned Hydrogel	Cancer therapy	Cancer treatment			Cytokines [131]
	GelDA-PDA-PPy Hydrogel	Cardiac repair	Enhanced conductivity and tissue adhesion	Enhanced osteogenesis capacity	Limited clinical efficacy	Astragaloside IV, Gelatin [103]
	Gel-pBP@Mg Hydrogel	Myocardial infarction repair	Enhance adhesion and controlled release	N.D.	N.D.	Magnesium, Polysaccharides [108]
	Gel-pBP@Mg	Myocardial infarction repair	Stabilizes BPNSs	Enhances material stability	Reacts with oxygen	Magnesium (Mg), Black Phosphorus Nanosheets (BPNSs) [134]
	TPQGel	Bone defect repair	Provides antioxidant properties	Antioxidant properties	Limited mechanical properties	Tri-calcium Phosphate (TCP), QK peptide [135]
**Sheet/Coating**	PDA-rGO electrode	Cardiac repair, self-powered sensor	Bioelectrical stimulation, mechanical energy harvesting	Self-powered, enhanced electroactivity	Increased sheet resistance	Reduced graphene oxide (rGO) [136]
	PDA@LDHs	Bone regeneration, drug release	Enhancing drug release and scaffold strength	Improved mechanical strength, controlled drug release	Potential burst release of drugs	LDHs, DMOG, eugenol [137]
	PDA-heparin modified sponge	Whole blood autotransfusion, anticoagulant	Anticoagulation, blood coagulation factor inactivation	Efficient anticoagulation, rapid sorption	Potential for side effects	Heparin-mimetic polymers (HMP) [138]
	PDA melanin-like pigment	Surface biofunctionalization, pigment coating	Progressive assembly, surface modification	Surface adhesion, NIR-to-heat conversion	Adhesive, prone to uncontrolled coating	PAINT initiator-loaded template [139]
	PDA@MS and B/PDA@MS	Bone regeneration, stem cell therapy	Improves sEV loading and release	Enhanced sEV loading, optimized release	N.D.	PDA, CaP [140]
	MFO coating	Osseointegration in RA, inflammation modulation	Regulates ROS, mitochondria dynamics, Ca^2+^ overload	Improved osteoimmunomodulation, M2 polarization	N.D.	MnFe_2_O_4_ nanoparticles, TiO_2_ [141]
	Sheltered positive charge polymeric coating	Antithrombotic applications, blood-contacting devices	Prevents surface-induced coagulation activation	Prevents thrombogenesis, avoids interfering with hemostasis	Still requires optimization for some clinical applications	Polymer (SpCM), PEG (polyethylene glycol), PDA [142]
**Fiber**	N.D.	Piezoelectric energy harvesting and sensing	Enhances interfacial adhesion and piezoelectricity	Multifunctional energy harvesting	Challenges in interfacial compatibility	Barium titanate (BTO), Polyvinylidene fluoride (PVDF) [117]
	Corn protein fiber	Wound healing monitoring, strain sensing	Forms conductive sensing layer	Versatile adhesion	Limited stability	Silver [143]
	PDA-mSF composite patch	Periodontal tissue regeneration in diabetes	ROS scavenging, inflammation modulation	Anti-inflammatory, promotes periodontal regeneration	Limited specificity in targeting	Metformin-ZIF system [144]
	Fe_3_O_4_@PDA hydrospongel	Localized drug delivery, tumor ablation, chemotherapy, magnetothermal therapy	Enhances drug release, photothermal properties	Enhanced drug delivery, tumor-targeted therapy, high mechanical stability	Potential iron toxicity, limited degradation rate	Fe_3_O_4_ nanoparticles, cellulose nanofibers, PDA [145]
	PFS@AM/CeO_2_	Bone repair, inflammation reversal	Adhesion and loading of enzymes	Activates macrophage efferocytosis	N.D.	Apoptosis-mimetic CeO_2_ nanoenzymes [146]
	PLLA/CFO fiber	Wound healing via multibiophysical stimuli	Facilitates magnetic, mechanical, and electrical stimulation	Enhanced interfacial coupling	Nondegradability	CFO nanoparticles, PLLA (Poly(lactic acid)) [116]

### 4.4. Polydopamine Nanoparticles

PDA nanoparticles are extensively studied nanomaterials because of their unique chemical structure. It has been reported that PDA nanoparticles are synthesized by self-polymerizing dopamine under normal circumstances and by a catecholamine derived from tyrosine [147,148]. Because PDA nanoparticles exhibit strong adhesion and endurance, they can be used in biomedical, environmental, and material engineering applications, providing nanomaterial advantages with the scalability of nanotechnology. PDA nanoparticles will be explored for their synthesis, properties, and potential applications, including drug delivery, imaging, biosensing, and other uses. Furthermore, it summarizes the current trends in this field and discusses the merits and challenges involved in the application of these nanoparticles [149,150]. An alkaline solution is used to oxidize and polymerize dopamine molecules to generate PDA nanoparticles. The oxidation of dopamine generates intermediates, such as dopamine quinones, which can be converted into polymer networks through self-polymerization. After this polymerization process occurs, the PDA becomes black, stable, and adhesive [151,152].

Several methods exist to modify PDA nanoparticles, including adjusting the dopamine content, reaction time, and solution pH [153,154]. Surfactants or other additives can be used to adjust the particle size and reduce adhesion. PDA nanoparticles range in size from nanometers to several hundred nanometers, and their shape can be irregular or spherical, depending on the synthesis conditions [155,156]. Different chemical groups or biomolecules are attached to the PDA nanoparticle surface to modify its characteristics. NPs are often modified to enhance targeting, improve biocompatibility, and increase endurance in biological environments through this surface treatment technique. PDA nanoparticles are highly efficient and versatile owing to their ability to modify surfaces [157,158,159].

Owing to its robust structure and surface-active functional groups, PDA is a highly versatile material that enables the formation of a wide range of nanostructures [160,161]. As an additional benefit, PDA acts as both a decreasing agent and a strengthening agent, enabling further processing for applications in biomedicine and nanotechnology [162,163,164]. As a result of these synthesis techniques, PDA nanoparticles have been demonstrated to possess adaptability and properties that make them candidates for various biomedical applications, including imaging, drug delivery, and tissue engineering.

### 4.5. Irregularly Shaped Nanoparticles

The use of PDA coatings on various substrates has gained significant attention across many industries. This coating is commonly used to overcome the limitations of other nanoparticles. Specifically, PDA has been utilized as a ‘gatekeeper’ for mesoporous silica nanoparticles (MSNs), which were previously loaded with cationic amphiphilic drugs, such as desipramine and doxorubicin, to block the nanoparticle pores. PDA functions as a pH-sensitive barrier, preventing a sudden burst of pore opening while enabling controlled drug release.

**Figure 10:**
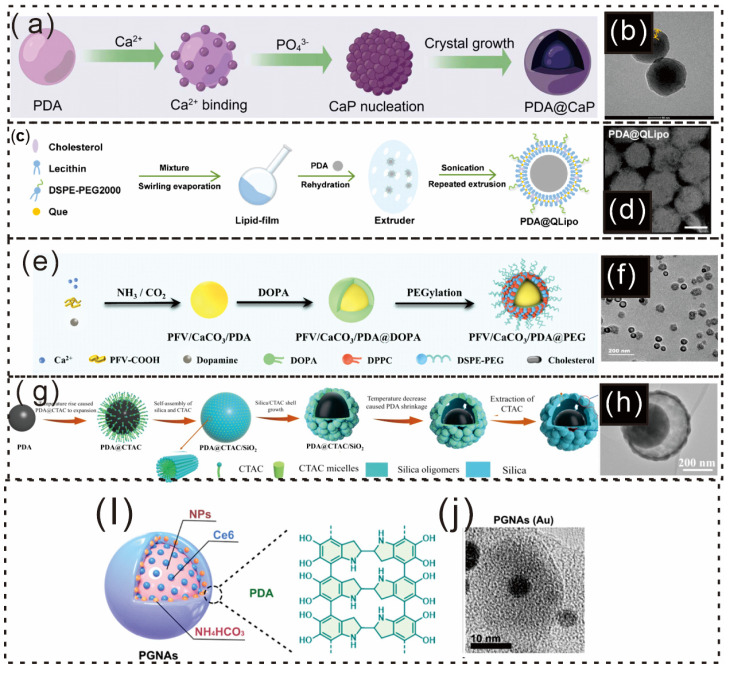
Schematic illustration and microscopic pictures of PDA-based nanoparticles. (**a**) Schematic representation of the biomimetic mineralization process of PDA@CaP, adapted from Figure Draw. (**b**) TEM image of PDA@CaP [165]. Copyright 2023, American Chemical Society. (**c**) Preparation of the PDA@QLipo nanosystem via a typical thin-rehydration method and uniform size distribution achieved via an extruder. (**d**) TEM image of PDA@QLipo [168]. Copyright 2024, American Chemical Society. (**e**) Schematic illustration of PFV/CaCO_3_/PDA@PEG nanoparticles. (**f**) TEM image of PFV/CaCO_3_/PDA@PEG [169]. Copyright 2024, American Chemical Society. (**g**) Fabrication of PDA@SiO_2_ nanocomposites with asymmetric yolk@shell and multisized pore structures. (**h**) TEM images of 60-PDA@SiO_2_−V synthesized with various volumes of CTAC solution (0.3) [170] Copyright 2024, American Chemical Society. (**i**) Schematic detailing the nanostructures of the prepared PGNAs. (**j**) TEM image of prepared PGNAs (Au); scale bar, 10 nm [12]. Copyright 2024, American Chemical Society.

Lu et al. prepared PDA nanoparticles, which were then suspended in Dulbecco’s modified Eagle’s medium (DMEM) before adding CaCl_2_ [87]. The mixture was incubated, with the catechol groups on the PDA nanoparticles binding to Ca^2+^ ions, providing the necessary nucleation sites for CaP formation (Figure 10a) [165]. As illustrated in Figure 10b, the resulting PDA@CaP formed in the 2-h reaction system displayed a core–shell structure and an average diameter of 150 nm [165].

QLipo was prepared by mixing quercetin (Que), cholesterol, lecithin, and DSPE-PEG in dichloromethane via a thin-rehydration method [166,167]. Yang et al. subsequently created PDA@QLipo by combining PDA with QLipo and then used sonication and membrane filtration to achieve consistent nanosystem sizes (see Figure 10c) [168]. The morphology, hydrodynamic diameter, and zeta potential of PDA, Lipo, QLipo, PDA@Lipo, and PDA@QLipo were characterized via TEM (Figure 10d), which revealed that PDA@QLipo had a particle structure with a uniform size [168]. Additionally, He et al. combined extracellular Ca^2+^-mediated antimetastasis with PDT and ROS-triggered calcium overload for synergistic skin tumor therapy via conjugated polymer–calcium composite nanoparticles (PFVs/CaCO_3_/PDA@PEG) (Figure 10e) [169]. SEM images (Figure 10f) revealed that PFV/CaCO_3_/PDA@PEG had a uniform hollow structure with an average diameter of 70 ± 10 nm [169].

Additionally, Wang et al. assembled PDA@cetyltrimethylammonium chloride (PDA@CTAC) composite microspheres via an “expansion−shrinkage” method and monomicelle interfacial confined assembly (see Figure 10g) [170]. Yolk@shell T-PDA@SiO_2_−V structures were synthesized at various reaction temperatures (50, 60, 70, and 80 °C) and CTAC volumes (0.1, 0.2, and 0.3 mL) [170]. TEM imaging revealed the structure of the prepared PDA@SiO_2_ yolk@shell hollow particles (Figure 10h) [170]. According to Chu et al., the generality of the constructed photoactivated gas-generating nanocontrast agents (PGNAs) was evaluated (Figure 10i), and three similar types of nanoparticles (SiNPs) were tested [12]. The TEM image revealed a distinct PDA shell surrounding the nanoparticles (Figure 10j) [12].

PDA-based particles offer remarkable potential for advanced applications in biomedical, environmental, and materials science because of their unique functional properties and ease of customization, as shown in Table 2. PDA coatings have emerged as versatile tools in drug delivery systems, offering controlled drug release and enhanced stability through pH-sensitive barriers. By integrating PDA with various nanoparticles and substrates, such as mesoporous silica and liposomes, PDA-based systems enable targeted therapies, including cancer treatment, skin tumor therapy, and brain drug delivery, demonstrating significant potential in biomedical and materials science applications.

There are several essential aspects to consider when evaluating gaps or limitations. First, the scalability of polydopamine-based fiber fabrication remains a challenge, as current methods may not readily lend themselves to large-scale industrial production owing to complex, time-consuming synthesis processes. Second, while polydopamine fibers offer excellent biocompatibility, their mechanical strength may not always be sufficient for certain applications, particularly in load-bearing environments [168]. Finally, although the versatility of polydopamine fibers is well established across various biomedical applications, their long-term stability under physiological conditions, including resistance to degradation and environmental factors, requires further investigation [170]. These limitations highlight the need for continued research to optimize synthesis methods, enhance mechanical properties, and improve the durability of polydopamine fibers for real-world applications.

### 4.6. Mesoporous Polydopamine

Mesoporous PDA is physiologically resilient. In addition to its ability to self-polymerize and form stable networks, this tendency also enhances its mechanical strength, which is vital for biomedical applications.

**Figure 11:**
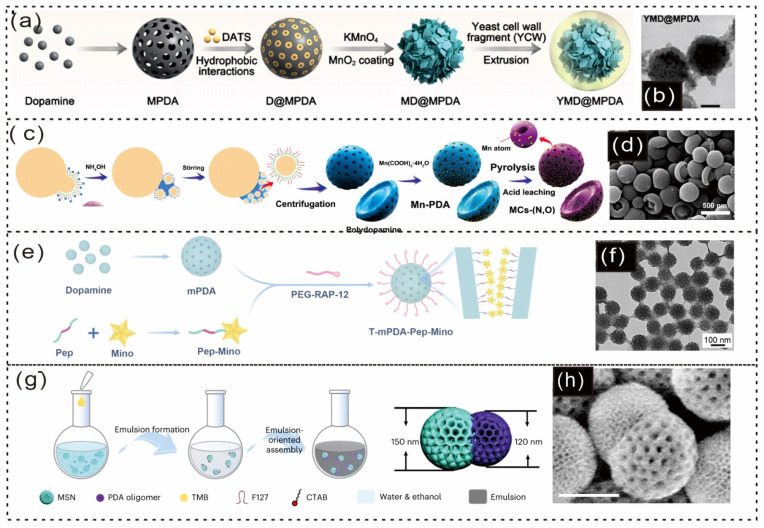
Schematic illustration and microscopic pictures of PDA-based mesoporous particles. (**a**) Schematic diagram illustrating the fabrication process of YMD@MPDA. (**b**) Transmission electron microscopy (TEM) image of MD@MPDA with a scale bar of 100 nm [66]. Copyright 2025, American Chemical Society. (**c**) Schematic representation of the fabrication process for MCs-(N,O). (**d**) Field emission scanning electron microscopy (FE-SEM) image of MCs-(N,O) [171] Copyright 2023, Wiley-VCH. (**e**) Schematic showing the synthesis of the TmPDA-Pep-Mino nanosystem. (**f**) TEM images of mesoporous nanoparticles [174]. Copyright 2023, Nature Portfolio. (**g**) Schematic of the emulsion-based assembly process for Janus double-spherical MSN&mPDA nanoparticles with tunable and large mesopores. (**h**) Dark-field TEM image of MSN&mPDA [175]. Copyright 2023, Nature Portfolio.

Xu Zhang and colleagues reported that a yeast-inspired, orally administered nanocomposite with responsive H_2_S release has been developed for the treatment of inflammatory bowel disease (IBD) [66]. First, the MD@MPDA core was fabricated by integrating manganese dioxide (MnO_2_) nanozymes onto DATS-loaded mesoporous PDA nanoparticles, referred to as MD@MPDA NPs. These NPs were then coated with a yeast cell wall (YCW) shell, resulting in the formation of YMD@MPDA NPs (Figure 11a), as shown in the TEM images in Figure 11b [66]. Additionally, Wang et al. synthesized atomic Mn-embedded, bowl-like mesoporous carbon particles with Mn–N_4_ configurations and C-O-C functional groups via a versatile nanoemulsion assembly method. A schematic of the formation process is shown in Figure 11c, and an FE–SEM image is shown in Figure 11d [171]. In another study, Di Wu and colleagues reported a pathogenesis-adaptive drug delivery system (DDS), pathogenesis-adaptive DDS (T-mPDA-PepMino), designed for the sequential regulation of ROS accumulation and microglial polarization in dynamic neuroinflammation, specifically for ischemic stroke therapy [172,173]. The T-mPDA-Pep-Mino nanosystem is based on mesoporous PDA functionalized with a minocycline-conjugated MMP-2-responsive peptide and a PEGylated brain-targeting peptide (Figure 11e) [174]. TEM images of this system are shown in Figure 11f [174].

Tiancong Zhao and colleagues reported that Janus mesoporous silica nanoparticles and PDA (MSN&mPDA) nanoparticles were created through emulsion-oriented assembly using 1,3,5-trimethylbenzene (TMB), Pluronic F-127, CTAB, and PDA on premade MSNs (Figure 11g) [175]. SEM images revealed that each MSN featured one mPDA hemisphere (~120 nm) with a radial mesopore channel, resulting in a double-spherical Janus MSN and mPDA nanoparticle (Figure 11h) [175]. PDA-based mesoporous particles exhibit excellent loading capacity, targeted delivery, and surface functionalization, making them applicable in medicine, as shown in Table 2. Mesoporous PDA nanoparticles provide outstanding mechanical strength, stability, and versatility for biomedical uses. Their ability to self-polymerize and modify surfaces improves drug delivery systems, making them suitable for targeted and responsive treatments. These features make PDA-based materials valuable tools for personalized medicine and disease management.

Mesoporous polydopamine nanoparticles, particularly in their Janus or dual-mesoporous forms, hold significant potential for various applications, such as drug delivery and catalysis. However, several key limitations must be addressed. One major issue is the restriction in pore size, with many mesoporous structures exhibiting pores smaller than 3 nm, which limits their capacity to load larger biomolecules or functional materials essential for advanced applications [171]. Additionally, while Janus nanoparticles are promising, achieving uniformity in pore size and compartment formation across the entire nanoparticle remains a challenge. This variability can affect the consistency and reliability of nanoparticles, especially in complex applications such as biological logic gates. Moreover, the synthesis of these advanced mesoporous structures requires highly controlled conditions, such as specific surfactant concentrations and emulsion systems, which complicates their scalability and makes large-scale production difficult [175]. Overcoming these challenges through more refined synthesis techniques and better pore control could unlock broader applications for mesoporous polydopamine nanoparticles in industrial and biomedical fields.

### 4.7. Polydopamine Spheres

PDA spheres are spherical nanoparticles formed by the self-polymerization of dopamine and are known for their high surface area and strong adhesion. These spheres can be easily functionalized with biomolecules for targeted drug delivery, imaging, and diagnostic applications. Owing to their versatility, they are widely utilized in medical applications.

**Figure 12:**
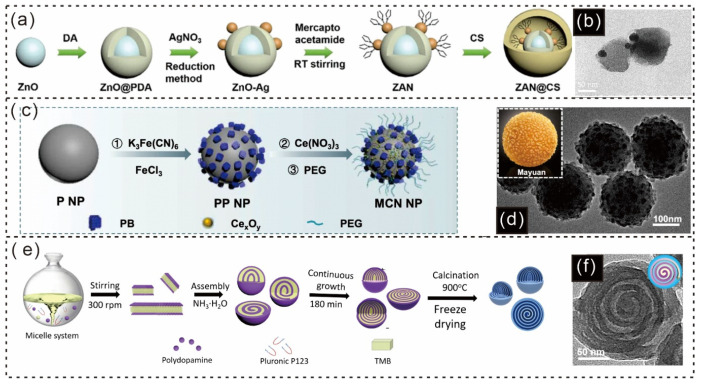
Schematic illustration and microscopic pictures of PDA-based nanospheres. (**a**) Schematic illustration of the fabrication process for ZAN@CS MNDs. (**b**) TEM images of ZnO-Ag [176]. Copyright 2023, Wiley-VCH. (**c**) Diagram illustrating the synthesis process of the MCN. (**d**) TEM images of MCNs; scale bar: 100 nm [64]. Copyright 2024, Nature Portfolio. (**e**) Schematic representation of the formation process for spiral MCNs, with lamellar micelle formation achieved by stirring the reactant at 300 rpm. (**f**) TEM image of spiral MCNs [56]. Copyright 2021, American Association for the Advancement of Science (AAAS).

Cong Liu and colleagues developed protonated charge reversal nanodrugs, namely, ZnO-Ag-mercaptoacetamide@chitosan (ZAN@CS) mesoporous nanodots (MNDs) (Figure 12a) [176]. The mucosal penetrability of ZAN@CS MNDs was demonstrated by assessing the viability of *H. pylori* colonized beneath the mucosa. The results indicated that ZAN@CS MNDs exhibited more potent antibacterial activity than ZAN MNDs did (Figure 12b) [176].

Additionally, MCN was prepared by Yamei Liu and colleagues, who sequentially grew Prussian blue (PB) and CexOy on P nanoparticles and subsequently modified the nanoparticles with polyethylene glycol (PEG) (Figure 12c) [64]. MCNs exhibited a unique structure similar to that of Mayuan, a traditional Chinese delicacy, due to the in situ growth of PB and Ce_x_O_y_ (Figure 12d, inset) [64].

In another study, Liang Peng and colleagues introduced MCNs that were fabricated via a lamellar micelle spiral self-assembly strategy with Pluronic P123 as the template, TMB as a hydrophobic mediator, and DA as the nitrogen and carbon source in an ethanol/water mixture (Figure 12e) [56]. The magnified TEM images revealed that the multishelled structure grew spirally from the particle center to the outer surface, with a continuous geometry and a clasp ring at the end (Figure 12f) [56]. PDA-based spherical particles offer outstanding stability, tunable surface properties, and high functionalization potential, making them highly effective in biomedical applications, as Table 2 also discusses several other studies. Owing to their high surface area, strong adhesion, and potential for functionalization, PDA spheres are highly effective for targeted drug delivery, imaging, and diagnostics. Their ability to form stable, adjustable nanoparticles allows for diverse applications in biomedicine, including antibacterial treatments, cancer therapy, and controlled drug release. These features make PDA spheres essential in advanced medical and therapeutic technologies.

While polydopamine spheres offer significant advantages in their tunable surface properties and potential for biofunctionalization, they also have notable limitations. First, the synthesis process of polydopamine spheres often requires precise control over the reaction conditions, such as pH and temperature, to achieve a uniform size and morphology [176]. Variations in these parameters can lead to inconsistencies in particle size distribution and surface characteristics, affecting reproducibility and scalability. Second, the mechanical properties of polydopamine spheres, including their stability under various environmental conditions, remain a challenge [64]. The relatively soft nature of these spheres can limit their application in high-stress environments or where structural integrity is critical. Finally, the biodegradability and long-term stability of polydopamine-based materials in vivo require further investigation, particularly regarding their breakdown products and potential cytotoxicity under prolonged exposure [56]. Addressing these issues will be essential for advancing the practical applications of polydopamine spheres in drug delivery and bioengineering.

### 4.8. Polydopamine Hollow Capsules

Recently, many applications, especially in biomedical areas, have utilized PDA hollow capsules. The coating templates make these capsules with PDA, and then, the core is removed. In addition to accurately shaping the capsules, the templates—whether soft or hard—are crucial for defining the structure.

**Figure 13:**
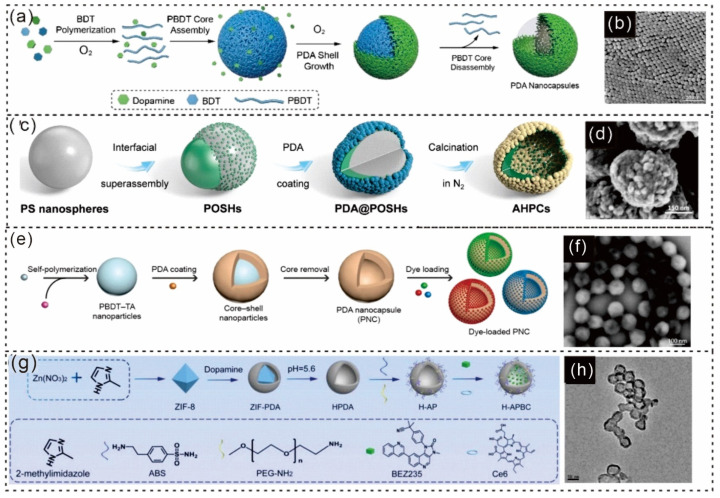
Schematic illustration and microscopic pictures of PDA-based capsules. (**a**) Redox-mediated pathway for the synthesis of core–shell and nanocapsule nanostructures. (**b**) SEM image of PBDT@PDA nanoparticles [177]. Copyright 2021, Wiley-VCH. (**c**) Schematic illustration of the preparation of POSHs, PDA@POSHs, and AHPCs. (**d**) SEM image of AHPCs [178]. Copyright 2022, Wiley-VCH. (**e**) Schematic diagram of the simple synthesis of dye-loaded PNCs using a supramolecular template (PBDT-TA). The glue-like nature of PDA enables the loading of small-molecule dyes (e.g., NB, AA, and MB) into PNCs via multiple interactions. (**f**) SEM image of PNCs [179]. Copyright 2022, American Chemical Society. (**g**) Schematic illustration of the synthesis methods for the H-APBC drug delivery nanoplatform. (**h**) TEM images of H-APBC [180]. Copyright 2022, SpringerLink.

Jiajing Zhou and colleagues reported that a redox-mediated kinetic strategy has been developed to synthesize uniform PDA nanocapsules using two simple molecules: dopamine and benzene-1,4-dithiol (BDT) [177]. A schematic of the formation process and the SEM image are shown in Figure 13a,b [177]. Similarly, Lei Xie described the formation of PDA@Janus polystyrene and organosilica hybrid nanoparticles (PDA@POSHs), and SEM images of these materials are presented in Figure 13c,d [178].

Another study by Wonjun Yim and colleagues discussed the formation of PNCs from PBDT-TAs, as shown in Figure 13e [179]. The rugged surface morphology of the PDA nanocapsule (PNC) is illustrated in the SEM images (Figure 13f). [179] Liu et al. synthesized hollow PDA (HPDA) nanoplatforms with a greater surface area and more internal space to facilitate drug loading. To optimize the biocompatibility and enable effective drug delivery, HPDA-ABS/PEG (HAP) was created by modifying ABS and PEG-NH_2_ on the surface of HPDA. BEZ235 and the photosensitizer chlorin e6 (Ce6) were encapsulated within HAP to form H-APBC nanocomposites (Figure 13g) [180]. A TEM image of H-APBC is shown in Figure 13h [180]. Hence, PDA-based capsules provide excellent structural stability, controllable release, and versatile surface functionality, making them highly suitable for targeted drug delivery and biomedical applications, as discussed in Table 2. PDA capsules, created by coating templates with PDA and then removing the core, provide exceptional structural stability and adaptable surface features. They are highly suitable for biomedical uses such as targeted drug delivery, thrombolysis, and phototriggered drug release. Owing to their ability to functionalize their surfaces and control release mechanisms, these capsules are promising options for sophisticated therapeutic systems.

Polydopamine (PDA)-based hollow capsules hold significant potential for various applications, but several limitations need to be addressed. The redox-mediated kinetic strategy for synthesizing uniform PDA nanocapsules shows promise; however, precise control of the size distribution and shell thickness remains challenging, as variations in reaction kinetics can lead to inconsistent shell uniformity [177]. Structural integrity is another concern, particularly for thinner PDA shells (under 10 nm), which may lack stability under harsh conditions, such as drying or high-pH environments, leading to collapse or deformation. Additionally, scalability remains a barrier, while the current synthesis method has been shown to work at larger scales, achieving high yield and consistent quality across large batches remains challenging [180].

### 4.9. Polydopamine Rods

PDA nanorods (NRs) are nanostructures created through dopamine polymerization that increase the mechanical strength and stability of the material. These rods possess strong adhesive properties, facilitating surface modification and functionalization across diverse applications. They are utilized in drug delivery, tissue engineering, and sensors because of their bioactivity and capacity to interact with biomolecules.

**Figure 14:**
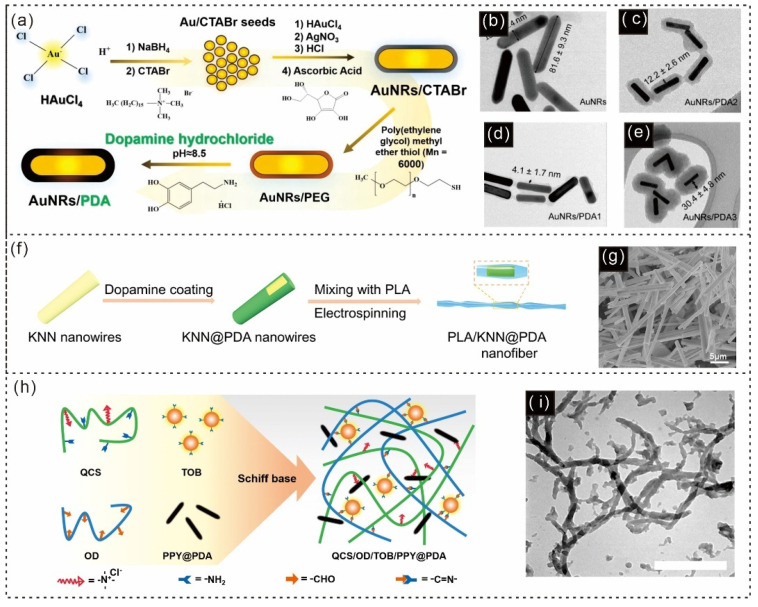
Schematic illustration and microscopic pictures of PDA-based nanorods. (**a**) Schematic representation of the synthesis of AuNRs/PDA, which consists of two main steps: the seed-mediated growth of AuNRs and the self-polymerization of PDA on the surface of the AuNRs. (**b**) TEM images of bare AuNRs, (**c**) AuNRs/PDA1, (**d**) AuNRs/PDA2, and (**e**) AuNRs/PDA3 [181]. Copyright 2023, Wiley-VCH. (**f**) Fabrication process of PLA/KNN@PDA nanofibers. (**g**) SEM image of KNN nanowires soaked in saline solution (pH 7.4, 37 °C) for 0 months [182]. Copyright 2022, American Chemical Society. (**h**) Diagram illustrating the formation of the QCS/OD/TOB/PPY@PDA hydrogel. (**i**) TEM image of PPY@PDA nanowires; scale bar: 500 nm [13]. Copyright 2022, American Chemical Society.

Daniel Aguilar-Ferrer, as illustrated in Figure 14a, described the synthesis of AuNRs/PDA, which involves two main steps. The first step is seed-mediated growth, followed by the second step, in which cetyltrimethylammonium bromide (CTABr) is replaced with PEG, allowing further self-polymerization of dopamine on the surface [181]. As expected, the thickness and density of the PDA shell can be controlled by adjusting the concentration of dopamine. Figure 14b–e show bare AuNRs and three Au/PDA composites with varying average shell thicknesses: 4.1 ± 1.7 nm (AuNRs/PDA1, Figure 14c), 12.2 ± 2.6 nm (AuNRs/PDA2, Figure 14d), and 30.4 ± 4.8 nm (AuNRs/PDA3, Figure 14e) [181].

Chen et al. proposed a 3D multichannel scaffold composed of piezoelectric PLA/KNN@PDA composite nanofibers for spinal cord repair via in vivo electrical stimulation (ES) [182]. This scaffold can act as a controllable electrical stimulator when activated by a programmable ultrasound (US) stimulus. The core component of piezoelectric scaffolds is electrospun poly(lactic acid)/potassium-sodium-niobate@PDA (PLA/KNN@PDA) nanofibers (Figure 14f) [182]. As shown in Figure 14g, the surfaces of the KNN nanowires exhibited noticeable physical changes over time, with the KNN nanowires cracking into smaller fragments [182].

Additionally, Ying Huang and colleagues reported that PDA-coated polypyrrole nanowires (PPY@PDA NWs) were uniformly dispersed in the hydrogel through hydrogen bonding with the QCS and OD [13]. These hydrogels, named QCS/OD/TOB/PPY@PDAn, where “n” indicates the concentration of PPY@PDA in mg/mL, were prepared as shown in Figure 14h [13]. The TEM image in Figure 14i confirms the successful preparation of the PPY@PDA NWs. PDA nanorods (NRs) possess remarkable mechanical strength, bioactivity, and surface modification capabilities, making them highly adaptable for biomedical uses. Their applications in drug delivery, tissue engineering, and sensors show strong potential. These nanostructures serve as dependable platforms for innovative therapeutic and diagnostic solutions.

**Table 2:** PDA nanoscale materials, their applications, and other components used in them.

	Name	Size/ Diameter	Applications	Function of PDA	Advantages	Disadvantages	Other Functional Components
Particles	Pt@PDA nanobowls	220–270 nm	Thrombolytic therapy	Photothermal conversion	Excellent catalytic performance	Limited motion under certain conditions	Platinum nanoparticles (Pt), PC liposomes [183]
	ZIF-8@PDA NPs (MOF PDA nanoparticles)	109.08 ± 0.8 nm	RNAi delivery, pest control (*S. frugiperda*)	Protects dsRNA from degradation, enhances uptake	Enhanced stability of dsRNA, synergistic effects with gut bacteria	Limited efficiency in some pest species	ZIF-8 (zeolitic imidazolate framework), dsRNA (double-stranded RNA) [184]
	ICPs@PDA/CuO_2_ NPs	116.45 ± 18.32 nm	Tumor therapy with PTT, CDT, and CT	Nanoparticle stability and drug delivery	High therapeutic efficiency, tunable ratio of drugs	Limited PTT efficiency at deeper tumors	PDA-Fe, CuO_2_, DOX, Gossypol [185]
	CMPBC (Cisplatin-loaded MSN/PB@CWL)	180 nm	Targeted drug delivery, cancer therapy	Self-thermophoretic propulsion, gas generation	Improved drug delivery efficiency, reduced side effects	Limited penetration in certain areas	MSN (mesoporous silica), PB (PDA-loaded nitric oxide donor), CWL (Lactobacillus rhamnosus GG cell wall) [186]
	AGPDA nanoparticles	~53.1 nm to 210 nm	Vaccine delivery, antigen presentation	Antigen presentation, immune modulation	Enhanced immune response, simple preparation	Limited clinical validation	miRNA, antigenic proteins [187]
	FMn@PMS	~280 nm	RA treatment, drug delivery	Cartilage adhesion, drug release	Improved joint retention, ROS response	Degradation in non-ROS conditions	MnO_2_, rapamycin [188]
	PDA@EM	83.5 ± 6.7 nm	Cancer diagnostics, subtype discrimination	Fluorescence quenching and restoration mechanism	High sensitivity, rapid profiling	Fluorescence quenching limitations	Erythrocyte membranes, fluorescent proteins [189]
	PDA-BP-ZnO composite	N.D.	Antibacterial, medical implants	Enhance corrosion resistance, antibacterial properties	Improved biocompatibility, antibacterial effect	Photothermal effect degradation under prolonged use	Black Phosphorus, Zinc Oxide [190]
	PDA-CRISPR–Cas system	~200 nm	Tumor gene therapy, gene editing	Tumor targeting, gene delivery	Deep tissue penetration, precise gene editing	Potential immune response, stability	CRISPR–Cas system [191]
	SPzyme	Diameter ~1 μm (spores)	Colitis treatment, ROS scavenging	Enhance spore germination	ROS scavenging, precise targeting	Limited to colitis treatment	Palladium nanoparticles, spore nutrient germinant [192]
	AGPDA, APDA	~53.1 nm to 210 nm	Atherosclerosis treatment, miRNA delivery	Antioxidant, drug delivery, MRI contrast	Enhanced ROS scavenging, biocompatibility	Limited stability in harsh environments	Gadolinium (Gd^3+^), Arginine (Arg), miR-146a [193]
	BaSO_4_@PDA@CeO_2_/DSP (BPCD)	N.D.	IBD treatment, CT imaging	Scavenges ROS, delivers drugs	Reduced ROS, targeted drug delivery	Toxicity risk, system complexity	CeO_2_, DSP [194]
	Tm@PDA-GA	131.8–5.6 nm	TNBC therapy, immune response	Drug loading, targeting	Enhanced immune response, drug penetration	N.D.	4T1 cell membrane, GA [195]
	PDA (pD) and polynorepinephrine (pNE) coatings	200 nm	Drug delivery, stabilization of nanocrystals	Surface functionalization, stabilization	High grafting density, stability improvements	Rapid clearance, liver uptake	PEG, polycatecholamines [196]
	CuS@PDA	236.9–11.1 nm	Biofilm Elimination, Antibacterial	Photothermal Conversion	Biofilm Disruption, Antibacterial, Synergistic Effect	Limited by NIR Absorption Spectrum	KG7 Peptide, CuS Nanoparticles [197]
	PDA Nanoparticles (PDNPs)	197.5–8.4 nm	Neuron and Myotube Activity Modulation	Photothermal Activation	Biocompatibility, Biodegradability, Antioxidant Properties	Potential Oxidative Stress During NIR Irradiation	Catechol, Quinone Reactive Groups [198]
	i-crystal	200 nm thickness	Controlled insulin release	Regulate insulin release	High insulin loading, prolonged release	Potential low stability over long-term use	Poly-_L_-lysine, FPBA microdomains [199]
	PtHD (Platinum-hyaluronic acid-poldopamine nanoparticle)	100.1 ± 3.0 nm	Gouty arthritis treatment, inflammation control	Catalytic activity for urate removal, photothermal effects	Multimodal therapy: urate removal, macrophage reprogramming, inflammation control	Potential immune response issues, complex system to produce	Platinum nanoparticles, hyaluronic acid, liposomes, M2 macrophage exosomes [200]
	Lv/Hb-PDA	50–200 nm	Tumor-targeted drug delivery	Facilitates self-assembly	Enhanced colloidal stability	Limited degradation	Lovastatin, Hemoglobin [124]
	PCN-DOX@PDA	N.D.	Tumor diagnosis, drug delivery	Photothermal agent, drug release	Enhanced absorption properties, High biocompatibility	Limited size control, Potential for toxicity	Fe-MOF, Doxorubicin [201]
	PLNP@PDA@DMMA/DOX	17.37 ± 1.57 nm	Tumor imaging, chemo-PTT therapy	Tumor-targeting, stability	Enhanced stability, Functional versatility	Limited drug loading, Potential cytotoxicity	Doxorubicin, DMMA, PEG [202]
	Pt_0.8_Co_0.2_@NC	~3.43 nm	Oxygen reduction, catalyst	Prevent Pt leaching, enhance stability	Enhanced stability, Antipoisoning ability	Reduced ORR activity with thick coatings	Nitrogen-doped graphene, Co [203]
	tBT@PDA-CPT NPs	N.D.	Tumor therapy, cell internalization	Enhance cell internalization	Biocompatibility, Biodegradability	Potential toxicity	Camptothecin (CPT), BaTiO_3_ [204]
	PDA@siBRAF/CaP	N.D.	Melanoma therapy	Enhance drug delivery	Enhanced drug delivery, Biocompatibility	Limited stability under neutral pH, Potential toxicity	Calcium phosphate (CaP) [165]
	Fe-BTC@PDA	N.D.	Pt adsorption	Enhance Pt recovery	Enhanced stability,	Limited regeneration efficiency	Thiol groups (DIP) [205]
	NDC Nanocages	N.D.	Tumor cell identification	Enhance SERS sensitivity	Biocompatibility	SERS signal suppression	Nitrogen doping (N) [206]
	CuSAE	N.D.	Tumor therapy	Enhance tumor penetration	High stability, Efficient internalization	Limited therapeutic activity	Glucose oxidase (GOx) [207]
	Co-SAEs/HNCS	N.D.	Cancer therapy	Enhance ROS generation	Photothermal conversion	Limited penetration depth	Hollow N-doped carbon sphere (HNCS) [208]
	PDA@SiO_2_ composite nanoparticles	Pore sizes: 15.4−86.5 nm	Cargo delivery, nanomotors	Multisized pores, tunable	On-demand drug delivery	Excessive porosity reduces integrity	SiO_2_ [170]
	PFV/CaCO_3_/PDA@PEG	N.D.	Antimetastasis, PDT	Calcium release, adhesion enhancement	High biocompatibility, efficient ROS generation	Limited stability under neutral pH	PFV, CaCO_3_, PDA, PEG [169]
	Pt−Ni nanoparticles	N.D.	Oxidase-like activity, biosensing	Oxidase-like activity	Robust antioxidants	Background signal interference	Nickel, Platinum [209]
	PDA@QLipo	N.D.	Hair regrowth, AGA treatment	ROS scavenging, angiogenesis	Biocompatibility, anti-inflammatory effects	Potential toxicity	Quercetin, Lipo [168]
	Cuf-TMB@PDA nanoparticles	N.D.	Antibacterial wound healing	Scavenges ROS, improves antibacterial efficacy	Enhanced antimicrobial properties	Limited long-term stability	Copper (Cu) [210]
	PD-G-MSNPs (Mesoporous Silica Nanoparticles)	~250–300 nm	Glutamine delivery for islet survival	Controlled nutrient release	Biocompatible	Potential toxicity	Glutamine (G), PD coating [65]
Mesoporous Structure	mPDA	N.D.	Scavenges ROS, inhibits neuroinflammation	Provides antioxidative properties	Efficient ROS scavenging	Precision treatment challenge	Minocycline (drug) [174]
	mPDA-SeMn-IR	228.3 ± 15.6 nm	Parkinson’s disease therapy	Antioxidant, photothermal, and neurostimulation functions	Simultaneous neuroprotection and modulation	Limited by the challenge of long-term treatment	SePh, MnO_2_, IR-1048 [211]
	Fe_3_O_4_@SiO_2_&mPDA	Diameter ~464 nm	Biofilm destruction, wound healing	Wetting behavior manipulation at the interface	Enhanced nanomotor properties, biofilm penetration	Limited to biofilm and wound healing	Magnetic nanoparticles, lanthanide fluorescent nanoparticles, Au nanorods [212]
	PDA-modified PLGA microscaffolds	~200 μm	Bone regeneration	Adsorption of sEVs	Enhanced loading efficiency	Limited release duration without biomineralization	CaP biomineralization [140]
	Mesoporous Carbon (MC)	N.D.	CO_2_ reduction	Modifies the electronic structure for reduced activation energy	Biocompatibility, Drug delivery	Toxicity concerns, Complex synthesis	Manganese (Mn) [171]
	(mPDA)	N.D.	Enhances antitumor immunity via photothermal therapy	Provides a photothermal effect	High biocompatibility	Instability in circulation	Salmonella-derived membrane vesicles [213]
	MSN&mPDA (Mesoporous Silica and PDA)	~150 nm (MSN); ~120 nm (mPDA)	Used in biological logic gates, drug delivery	Provides surface functionalization for logic gates	Biocompatibility, Targeted drug delivery	Limited loading capacity	No additional components [175]
	Mesoporous WO_3_	~180 nm	Sensing biomarkers for foodborne bacteria	Enhances gas-sensing properties	Biocompatibility, Drug delivery	Toxicity concerns, High production cost	Phosphorus (P) [214]
	Fe_3_O_4_@DMS&PDA@MnO_2_-SRF	170 nm	Boosting ferroptosis in tumor therapy	Synergizing GSH depletion and ferroptosis	Targeted drug delivery	Ferroptosis inhibition	MnO_2_, SRF [215]
Nano Sphere	AM/GW@PDA	N.D.	Immune modulation, hepatocellular carcinoma	Targeting exosome biogenesis and PD-L1 expression	Tumor-specific targeting, immune modulation	Limited release and degradation control	GW4869, amlodipine (AM) [216]
	MLS [185]	15 nm shell thickness	Cell protection, biocompatibility	Shell formation via dopamine oxidation	Enhanced protection, adaptability	Limited scalability of the process	*Saccharomyces cerevisiae*, alcohol oxidase, horseradish peroxidase [217]
	Dp825/ARS@PDA−Fe(III)−FA ICP NCPs	111.9 ± 37.1 nm	Tumor targeting and therapy	Stability, drug release	High drug loading, stability	Limited tumor penetration	IR825, DOX, FA, Fe(III) [218]
	PS@PDA-ICG	1 μm	Photothermal therapy, NIR imaging	Encapsulation, fluorescence enhancement	High biocompatibility, efficient propulsion	Complex fabrication, potential fluorescence quenching	ICG, PS core [219]
	PS@PDA-ICG	N.D.	Tumor photothermal therapy	Shell for fluorescence and propulsion	Active motion, real-time tracking	N.D.	ICG (fluorescent agent) [219]
	ZnO-Ag-mercaptoacetamide@chitosan (ZAN@CS)	N.D.	*H. pylori* eradication with gut microbiota protection	Active targeting, mucosal penetration	Targeting capability	Potential gut flora disruption	ZnO, Ag, mercaptoacetamide, chitosan [176]
	Synthetic melanin nanoparticles	70–500 nm	Mimic skin phototypes for biomedical optics	Light absorption and scattering for PA imaging	Good biocompatibility	Potential toxicity, Aggregation issues	None [220]
	PDA	N.D.	Bone repair and tumor treatment	Photothermal and prodrug release	Drug delivery system	Potential toxicity, Limited stability	Pt(IV) prodrug [221]
Capsule	CuS@PPDA nanoplatform	220–255 nm	Biofilm disruption	Enhance adhesion, stability	High photothermal efficiency, biofilm penetration	Reduced light absorption efficiency	CuS nanoparticles [197]
	Gold Nanorods (AuNRs)	Length: 81.6 ± 9.3 nm, Diameter: 18.0 ± 2.4 nm	Tumor therapy, photocatalysis, sensing	To suppress the cytotoxicity of CTAB, enhance the plasmonic properties	Targeted drug delivery	Possible aggregation	PEG, PEGylated graphene oxide, Rhodamine 123 [181]
	Fe_3_O_4_@Au Hybrid Nanorods	Fe_3_O_4_ NRs: 20 nm × 110 nm, Au NRs: 41 ± 4 × 93 ± 8 nm	Magnetic alignment for photoacoustic imaging	Magnetic alignment, modulation of plasmonic excitation	Biocompatibility	Signal noise	PEG, cystamine [222]
Nano Rod	Au Nanorods, Au@Ag Nanorods	Au Nanorods: 41 ± 4 × 93 ± 8 nm	FRET efficiency enhancement, biosensing	Self-assembly, FRET enhancement	Biocompatibility	Limited spectral tuning flexibility	PEG, Au@Ag [223]
	Gold Nanorods (GNRs)	Length: 54 ± 2 nm, Diameter: 15 ± 1 nm	Tumor therapy (chemo-thermal therapy)	To suppress the cytotoxicity of CTAB, high cisplatin loading, stable iodine-125 labeling	Targeted delivery, Stability enhancement	Potential cytotoxicity	PEG (Polyethylene glycol), Cisplatin, RGD peptides, Iodine-125 [224]

**Abbreviations:** Multifunctional nanorobot structure (MF-NRS), fluorescent nanodiamonds (FNDs), pH dual-response multifunctional system (PCN-DOX@PDA), ovalbumin (OVA), ceria nanoparticles (CeONPs), tetradecanol (PCM), single domain (SD), degradable metallic complexes (PtH@FeP), persistent luminescence nanoparticles (PLNPs), nitrogen-doped graphene (NC), oxygen reduction reaction (ORR), tetragonal BaTiO_3_ (tBT), camptothecin (CPT), serine/threonine protein kinase BRAF (BRAF, where RAF stands for rapidly accelerated fibrosarcoma), MOF (Fe-BTC), nitrogen-doped carbon (NDC), surface-enhanced Raman scattering (SERS), copper single-atomnanozyme (CuSAE), hydrophilic dendrimer (PAMAM), cobalt single-atom enzyme (Co-SAE), hollow N-doped carbon sphere (HNCS), hyaluronic acid (HA), organic framework polydopamine heterostructure (MOF-PDA), poly(fluorene-*co*-vinylene) (PFV), imiquimod (IQ), copper-based nanoparticles called polydopamine (PDA)-coated copper-amine (Cuf-TMB@PDA), polydopamine (PD), glutamine (G), sorafenib (SRF), dendritic mesoporous silica (DMS), dopamine nanoscale coordination polymer (DA-NCP), ZnO-Ag mercaptoacetamide@chitosan (ZAN@CS), janus polystyrene and organosilica hybrid nanoparticles (POSHs), gold nanorods (GNRs).

In vitro cytotoxicity experiments have shown PDA-based materials to be minimally toxic at the levels of interest and to support good cell viability across a variety of cell lines. In addition, in vivo experiments investigating clearance kinetics show that smaller PDA nanoparticles, generally within the nanorange, are cleared quickly by the renal filtration system. In contrast, larger particles are cleared slowly and can accumulate in organs like the liver and spleen. As far as long-term toxicology is concerned, past research indicates that the degradation products of PDA, such as catechol derivatives and quinones, are mostly biocompatible. However, more studies are needed to evaluate their long-term effects in the living environment, especially in cases involving larger PDA structures. All these data point to the possibility of using PDA-based materials in the biomedical context and, therefore, to the need for additional research in the clinical environment.

### 4.10. Other Types

**Figure 15:**
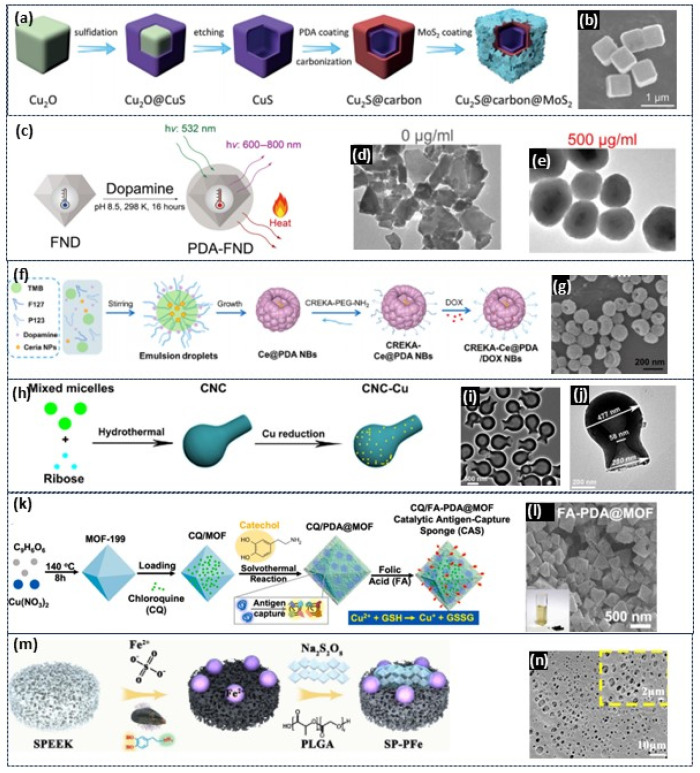
Schematic illustration and microscopic pictures of PDA-based composites. (**a**) Diagram illustrating the synthesis of three-layered Cu_2_S@carbon@MoS_2_ nanoboxes. (**b**) Visualization of Cu_2_S@carbon nanoboxes [225]. Copyright 2020, Wiley-VCH. (**c**) Schematic representation of dual-functionalized PDA-FNDs derived from FNDs. In this system, FNDs serve as luminescent nanothermometers, whereas PDA results in light-induced heat release. (**d**,**e**) Transmission electron microscopy (TEM) images of PDA-FNDs synthesized with various concentrations of dopamine hydrochloride solutions, as noted above each image. Image dimensions: 751 × 534 nm. The brightness and contrast were adjusted for enhanced visibility [226]. Copyright 2021, American Association for the Advancement of Science (AAAS). (**f**) Schematic diagram of the preparation of CREKA-modified Ceria@polydopamine nanobowls (CREKA-Ce@PDA NBs). (**g**) SEM images showing the Ce@PDA nanobowls [227]. Copyright 2023, American Chemical Society. (**h**) Schematic illustration of the CNC-Cu fabrication process. (**i**) TEM image displaying the structure of the CNCs. (**j**) Magnified TEM image providing detailed views of the CNC structure. [228] Copyright 2022, American Chemical Society. (**k**) Schematic diagram outlining the synthesis of CQ/FA-PDA@MOF. (**l**) SEM image of the FA-PDA@MOF material [229]. Copyright 2023, Elsevier. (**m**) Schematic illustration depicting the fabrication process for the SP-PFe implants. (**n**) SEM images revealing the surface characteristics of SPEEK [230]. Copyright 2025, American Chemical Society.

Yongjin Fang presented a meticulous design and fabrication approach for creating three-layered Cu_2_S@carbon@MoS_2_ hierarchical nanoboxes via a simple, multistep template-engaged strategy. The process begins with the use of Cu_2_O nanocube templates, followed by the formation of CuS nanoboxes through sequential sulfidation and etching steps (Figure 15a) [225]. FESEM images confirmed that the CuS@PDA nanobox structure was well preserved (Figure 15b) [225]. In another experiment, dopamine molecules were polymerized into PDA on the surface of fluorescent nanodiamonds (FNDs) in Tris-HCl buffer (pH 8.5), resulting in the formation of PDA-FNDs after centrifugation and washing (Figure 15c) [226]. The size, shape, and thickness of the PDA layers were assessed via transmission electron microscopy (TEM), which revealed that the FNDs possessed sharp edges and a broad distribution of sizes and shapes (Figure 15d,e) [226].

In a similar study, Minxia Zhu synthesized bowl-shaped ceria@polydopamine nanomotors, followed by an asymmetric coating with CREKA on the outer surface. The antitumor agent doxorubicin (DOX) was then loaded into the system. The ceria core catalyzes the conversion of endogenous H_2_O_2_ to O_2_, depleting tumor-specific H_2_O_2_ inside the nanobowls [227]. This generates a self-induced gradient that drives the motion of the nanomotors. Moreover, the outer CREKA coating reorients the nanomotor’s movement toward tumor cells via receptor—ligand interactions. This enhanced motion enables deeper penetration into tumor tissues and improves the cellular uptake efficiency compared with conventional tumor-targeting nanoparticles used in chemo-photothermal therapy (Figure 15f) [227]. As illustrated in Figure 15g, PDA nanobowls (NBs) and Ce@PDA NBs with well-defined openings were synthesized with an equal volume of TMB. Moreover, Ce@PDA nanospheres (NSs) with a regular spherical shape were produced when less TMB was added. SEM images revealed that both Ce@PDA NSs and NBs possess core–shell structures, with dark ceria cores and lighter PDA shells (Figure 15g) [227].

Inspired by self-propelled biological motors, such as enzymes, a near-infrared (NIR) light-driven nanomotor catalyst (CNC-Cu) was developed, consisting of a carbonaceous nanocalabash (CNC) core with a copper load for active targeted CuAAC, allowing for the localized synthesis of drugs [228]. The CNC-Cu nanomotor is designed to move at a controllable rate, actively targeting deep-layered biofilms (Figure 15h). SEM images revealed that the CNCs had a uniform size of approximately 500 nm, with a prominent open neck structure (Figure 15i,j) [228].

Chiang et al. detailed the synthesis of CAS, a composite of chloroquine (CQ), a copper-based metal–organic framework (MOF), and PDA nanogels (Figure 15k) [229]. The morphologies of FA-PDA@MOF and CAS were characterized via SEM and TEM (Figure 15l) [229].

In a study addressing the high failure rates of bacterial-induced bone defect restorations and complications from secondary infections, an orthopedic implant was designed that incorporates Na_2_S_2_O_8_ and Fe^2+^ coatings on sulfonated PEEK (SPEEK) implants [230]. The process for constructing a modified coating on SPEEK implants is depicted in Figure 15l. FE–SEM was used to examine the micromorphology, and the surface of the SPEEK treated with concentrated sulfuric acid exhibited numerous porous structures, as shown in Figure 15m [230]. PDA-based nanostructures such as nanoboxes, nanomotors, and nanogels are rapidly emerging as versatile tools for drug delivery, biofilm targeting, and tumor treatment. Their distinctive features, such as self-propulsion and surface customization, improve therapeutic effectiveness and targeting accuracy. These developments highlight the significant potential of PDA nanomaterials in biomedical engineering and personalized medicine.

## 5. Applications

These materials are widely used in drug delivery systems, where the PDA component enables the controlled release of therapeutic agents, enhancing treatment efficacy and minimizing side effects. In tissue engineering, PDA composites provide a supportive microenvironment for cell adhesion and growth, making them ideal for scaffolds and implants in regenerative medicine. Additionally, their wound healing applications are significant, as they support tissue repair and speed up healing by promoting cell migration and angiogenesis.

### 5.1. Cancer Treatment

PDA nanoparticles can be modified with targeting ligands to direct therapeutic agents precisely to tumor sites, improving treatment effectiveness and minimizing side effects. Moreover, PDA naturally exhibits photothermal and photodynamic properties, making it an effective platform for combined cancer therapies with light-based methods.

**Figure 16:**
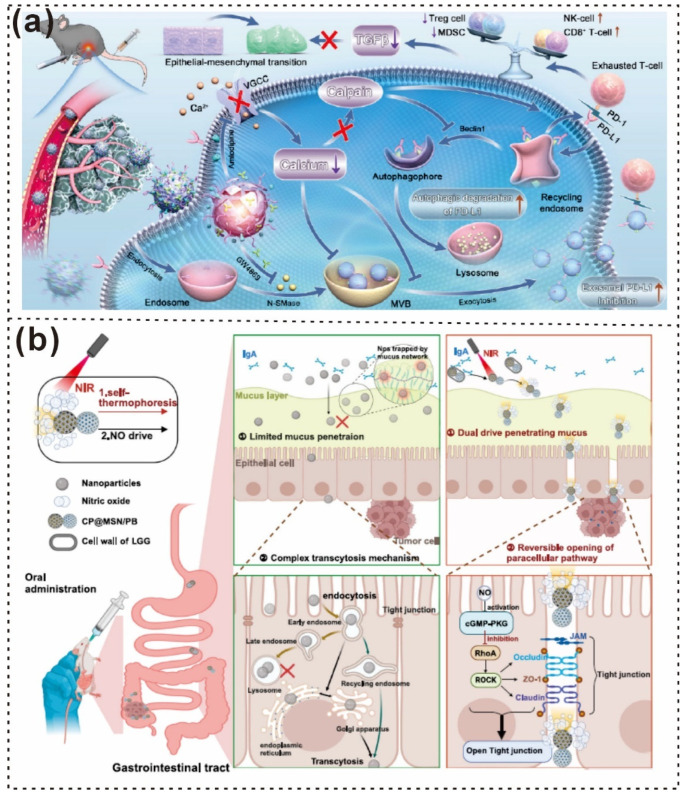
PDA applications in cancer treatment. (**a**) Schematic illustrations showing the dual-synergistic nanomodulator AM/GW@PDA, which induces the autophagic degradation of PD-L1 via calcium deprivation and disrupts exosome biogenesis and secretion. This process decreases the release of PD-L1-loaded exosomes into the tumor microenvironment (TME), resulting in favorable remodeling of the TME in hepatocellular carcinoma (HCC) [216] Copyright 2024, American Chemical Society. (**b**) A schematic comparison of nanoparticles (NPs) used for CRC treatment through passive diffusion versus dual-driven nanoparticles that improve the penetration of the intestinal mucus and epithelium [186]. Copyright 2025, Nature Portfolio.

Zhu and colleagues examined how the immunosuppressive tumor microenvironment (TME), generated by incomplete radiofrequency ablation (iRFA) in hepatocellular carcinoma (HCC), encourages tumor growth and dissemination [216]. They suggested a treatment strategy aimed at altering the post-iRFA TME by inhibiting exosome production, secretion, and PD-L1 expression, thereby reactivating cytotoxic T lymphocytes to slow the advancement and spread of HCC. By utilizing the versatile properties of PDA nanomodulators, they created a targeted delivery system for GW4869 and amlodipine (AM), enabling the tumor-specific release of these drugs (Figure 16a) [216].

Oral drug delivery systems inherently hold promise for the treatment of colorectal cancer. However, their effectiveness is often compromised by intricate intestinal barriers, notably mucus and the epithelium, leading to limited clinical application and subpar therapeutic outcomes. Wang et al. and their team designed a bioactive, self-thermophoretic nanomotor that is also gas-powered and capable of effectively penetrating both mucus and epithelial barriers for colorectal cancer therapy (Figure 16b). The capacity of PDA to engage with the tumor microenvironment and facilitate immune modulation expands its potential as a versatile therapeutic agent [186]. PDA composites have versatile uses in targeted cancer therapy because they enable precise drug delivery and influence the tumor microenvironment. The natural photothermal and photodynamic properties of these materials further increase their effectiveness in combined treatments. These features make PDA-based systems promising platforms for improving cancer treatment approaches.

### 5.2. Chemodynamical Therapy

Reactive oxygen species (ROS) enable PDA to intensify localized oxidative stress, leading to cell death and tumor regression. The biocompatibility and straightforward surface modification of PDA facilitate its use in targeted drug delivery, allowing it to be conjugated with therapeutic agents or nanoparticles to improve treatment outcomes.

**Figure 17:**
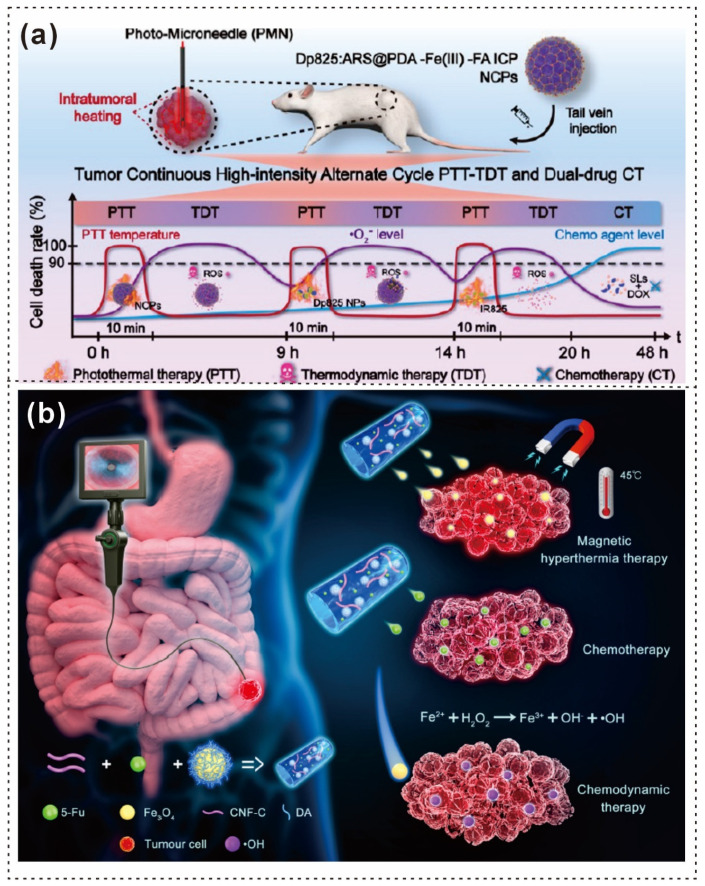
PDA applications in chemotherapy. (**a**) Schematic diagram of molecular stacking in infinite coordination polymer nanocomposites used for continuous, high-intensity photothermal−thermodynamic cycle therapy and chemotherapy against tumors [218]. Copyright 2025, American Chemical Society. (**b**) Schematic illustration of the use of HTHSG for treating CRC through magnetothermal, chemo-, and chemodynamic therapies [145]. Copyright 2025, Nature Portfolio.

To enhance the combined therapeutic effectiveness of tumor photothermal, thermodynamic, and chemotherapy treatments and overcome challenges such as limited laser penetration, uneven heating, and poor timing between methods, Chenyu Zhao and colleagues developed a photomicroneedle (PMN) system. This system incorporates an optical fiber into a perforated stainless-steel microneedle, enabling laser emission from both the tip and sidewalls for uniform intratumoral irradiation [218]. The PMN supports continuous high-intensity photothermal and thermodynamic alternating cycle therapy combined with chemotherapy, resulting in over 90% tumor cell death within 48 h, as shown in Figure 17a [218].

Preserving anal function in advanced-stage colorectal cancer (CRC) remains a key challenge. Wu and colleagues presented hollow-tube hydrospongel (HTHSG) as a novel neoadjuvant treatment. Made from cellulose nanofibers crosslinked with Fe_3_O_4_@PDA nanoparticles, HTHSG offers rapid swelling in approximately 10 s and a high fracture strength exceeding 250 kPa, ensuring mechanical robustness [145]. This hydrogel allows targeted, localized delivery of the chemotherapy drug 5-FU directly to the tumor. Additionally, in addition to traditional chemotherapy, HTHSG utilizes electromagnetic induction for focused thermal ablation and chemodynamic therapy, reducing damage to healthy tissues, as shown in Figure 17b. Moreover, the ability of PDA to absorb near-infrared (NIR) light enables its use in combination therapy, where heating from the NIR region can enhance its chemodynamic effects [145]. PDA enhances tumor therapy by producing reactive oxygen species (ROS), causing localized oxidative stress that leads to cell death and tumor shrinkage. New techniques, such as photomicroneedles and hollow-tube hydrogels, leverage the ability of PDA to be used for combined treatments, including chemotherapy and photothermal therapy. These innovations enhance targeted delivery, minimize side effects, and improve overall therapeutic results in cancer treatment.

### 5.3. Drug Delivery System

The inherent biocompatibility, simple synthesis, and surface modifiability of PDA make it a suitable choice for controlled, targeted drug delivery. PDA nanoparticles can be functionalized with diverse ligands, enabling the selective targeting of particular cells or tissues, which increases therapeutic effectiveness and reduces off-target effects.

**Figure 18:**
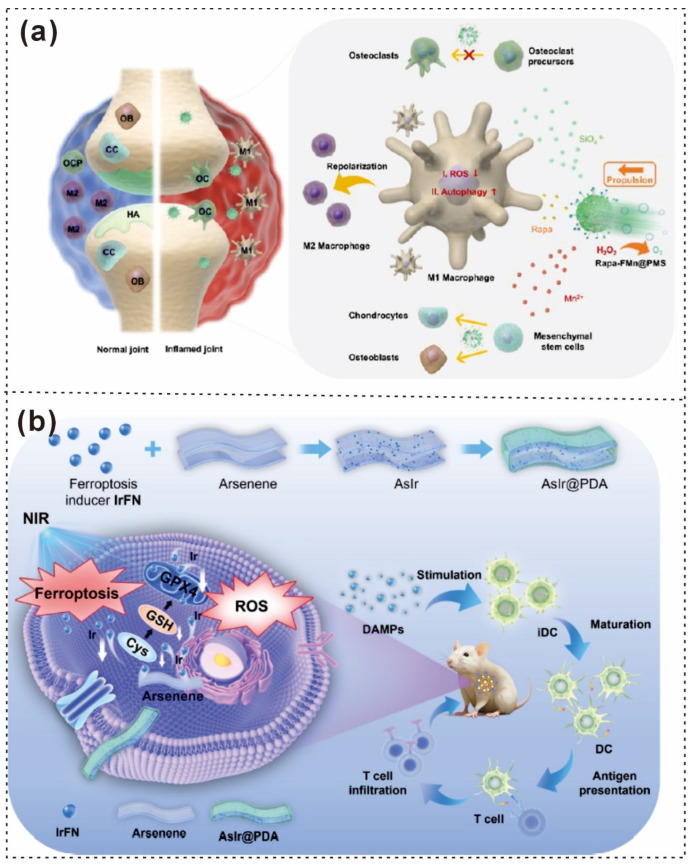
PDA as a drug delivery agent. (**a**) Schematic illustration of mesoporous silica-based nanomotors loaded with rapamycin (rapa-FMn@PMS) for synergistic rheumatoid arthritis treatment [188]. Copyright 2025, American Chemical Society. (**b**) Diagram showing the systemic delivery of AsIr@PDA for inducing ferroptosis-based chemotherapy and immunotherapy in pancreatic cancer [231]. Copyright 2024, Wiley.

Ma et al. explored the reduction in intracellular oxidative stress to balance M1 and M2 macrophage types, offering a promising approach for treating rheumatoid arthritis (RA). They created a hydrogen peroxide (H_2_O_2_)-activated nanomotor, FMn@PMS, which was explicitly designed to target M1 macrophages for the targeted delivery of rapamycin (Rapa) in RA therapy. These nanomotors were produced by growing manganese dioxide (MnO_2_) nanozymes directly on the surface of PDA-coated mesoporous silica nanoparticles, followed by surface modification with folic acid to ensure delivery to M1 macrophages (Figure 18a) [188].

Given the challenge of multidrug resistance and the high chance of recurrence, there is an urgent need for effective, less toxic treatments for pancreatic cancer. These cancer cells are notably resistant to apoptosis but remain vulnerable to ferroptosis. Zhao and colleagues developed an innovative nanoplatform, AsIr@PDA, created by electrostatically attaching a cationic iridium complex (IrFN) to two-dimensional arsenene nanosheets. This nanoplatform demonstrates enhanced ferroptosis-inducing capabilities with a high drug loading capacity and, importantly, effectively activates anticancer immune responses, leading to the efficient destruction of pancreatic tumors without any visible side effects, as shown in Figure 18b [231]. Furthermore, the capacity of PDA to strongly interact with a broad spectrum of drug molecules—both hydrophobic and hydrophilic—positions it as a promising carrier for various therapeutic agents.

### 5.4. Immune Modulation

Owing to its biocompatibility, biodegradability, and ability to interact with various biomolecules, PDA can modulate immune responses in a controlled manner. Studies indicate that PDA can influence the activation and polarization of immune cells, such as macrophages and dendritic cells, which play crucial roles in initiating and regulating immune responses.

**Figure 19:**
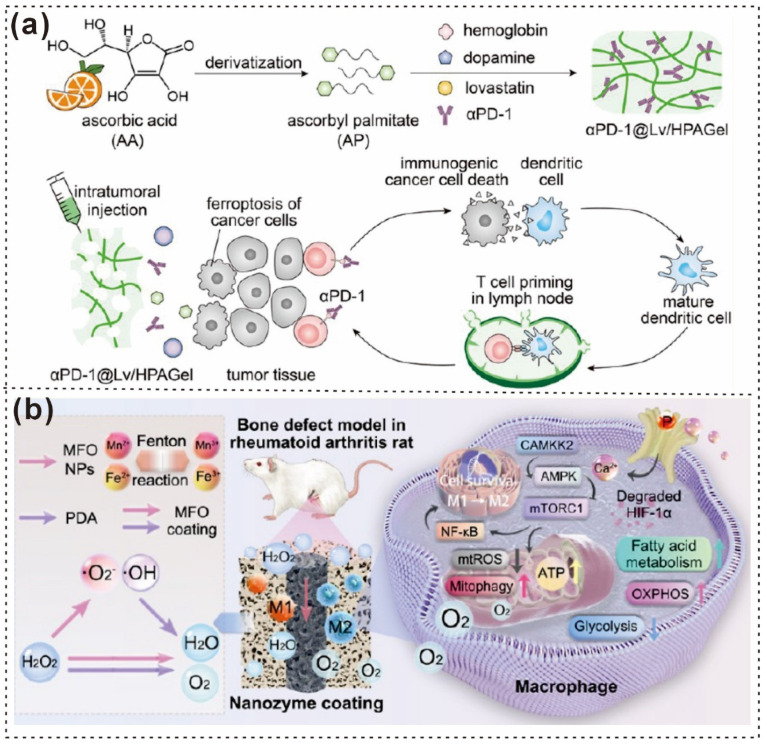
PDA applications in immune modulation. (**a**) Schematic illustration of supramolecular gels derived from ascorbic acid that trigger immunogenic ferroptosis in cancer cells, enhancing tumor immunotherapy [124]. Copyright 2025, American Chemical Society. (**b**) Schematic illustration of how nanozyme coatings induce mitochondrial metabolic reprogramming in macrophages to modulate immune responses for improved osseointegration in rheumatoid arthritis patients [141]. Copyright 2025, American Chemical Society.

According to Chen et al., traditional titanium (Ti) implants often fail to achieve osseointegration in bone defects associated with rheumatoid arthritis (RA), primarily because the RA microenvironment is characterized by high levels of reactive oxygen species (ROS) and hypoxia. This setting leads to mitochondrial dysfunction and intracellular Ca^2+^ overload, promoting the polarization of macrophages toward the M1 phenotype, which hinders osteoimmunomodulatory osseointegration. To address this, a nanozyme-inspired coating was created by depositing MnFe_2_O_4_ nanoparticles onto a PDA-decorated microporous TiO_2_ surface on Ti (the MFO coating). The osteoimmunomodulatory osseointegration of this coating was evaluated both in vitro and in vivo in the context of RA, and the underlying mechanisms were also investigated, as illustrated in Figure 19a [124].

High-dose ascorbic acid (AA) therapy mainly kills cancer cells through its oxidized form, dehydroascorbic acid (DHA). However, maintaining therapeutic AA levels inside tumors and overcoming hypoxia within tumors are significant challenges for the clinical use of AA. Cai et al. created an injectable supramolecular gel (αPD-1@Lv/HPAGel) made of ascorbyl palmitate (an AA derivative), lovastatin-loaded hemoglobin nanoparticles (Lv/Hb-PDA), and the immune checkpoint inhibitor anti-PD-1 (αPD-1). When injected into tumors, this gel maintains high local AA levels and helps convert AA into DHA by alleviating hypoxia through the release of oxygen from hemoglobin. Moreover, lovastatin blocks glutathione peroxidase 4, increasing AA-induced ferroptosis. This combined trigger of ferroptosis alters the tumor immune environment, triggering a robust immune response (Figure 19b) [141]. Additionally, PDA-based materials have been used to deliver immunomodulatory agents, providing a platform for targeted treatments for autoimmune diseases, cancer immunotherapy, and vaccine development.

### 5.5. Anti-Inflammatory Effects

The distinctive chemical structure of PDA, which features catecholic groups, enables it to interact with biological molecules, thereby modulating immune responses and alleviating inflammation. Research indicates that PDA can inhibit the activation of proinflammatory cytokines and enzymes, such as cyclooxygenase-2 (COX-2), both of which play crucial roles in inflammation.

**Figure 20:**
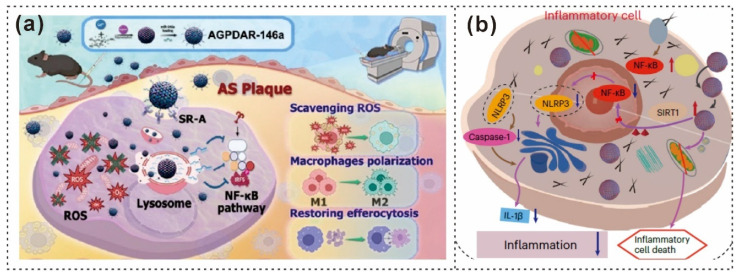
PDA applications in anti-inflammatory applications. (**a**) Schematic illustration of anti-inflammatory nanomedicine for atherosclerosis treatment targeting inflammatory macrophages delivered by miRNAs visible via MRI [193]. Copyright 2025, American Chemical Society. (**b**) Mechanisms of the anti-inflammatory therapeutic modality of D-N[EM2] [200] Copyright 2024, Nature Portfolio.

Atherosclerosis, a leading cause of cardiovascular diseases, is a chronic inflammatory condition. To address this, combining strategies to regulate oxidative stress and inflammation provides an effective treatment option. Li and colleagues developed a specialized nanosystem called PDA nanoparticles doped with arginine and gadolinium ions (AGPDAR-146a), which are designed to deliver therapeutic oligonucleotides—specifically microRNA-146a (miR-146a)—targeting inflammatory macrophages in atherosclerotic plaques [193]. These AGPDAR-146a nanoparticles efficiently load and transport miR-146a, increasing its accumulation in inflammatory macrophages through specific interactions between miR-146a and class A scavenger receptors (Figure 20a) [193].

Moreover, gouty arthritis is a persistent, progressive condition characterized by elevated urate levels in the joints and an inflammatory immune environment. Clinical evidence shows that treatments focusing solely on lowering urate or reducing inflammation often do not yield optimal results. Xu and colleagues created a sophisticated biomimetic nanosystem with a ‘shell’ made from a fusion membrane of M2 macrophages and exosomes [200]. This system encapsulates liposomes containing uricase, platinum-in-hyaluronan/polydopamine nanozymes, and resveratrol. It specifically targets inflamed joints, encouraging the local build-up of anti-inflammatory macrophages, whereas uricase and nanozymes work to lower urate levels within the joints (see Figure 20b) [200]. Additionally, PDA has been shown to stabilize bioactive molecules, increasing their therapeutic efficacy while minimizing side effects.

### 5.6. Photothermal Therapy

The distinctive optical properties of PDA, such as strong absorption in the near-infrared (NIR) region, make it an effective agent for converting light into heat, facilitating targeted cancer therapy. PDA nanoparticles are easy to synthesize and functionalize, enabling the creation of versatile delivery systems that increase therapeutic efficacy and reduce side effects.

**Figure 21:**
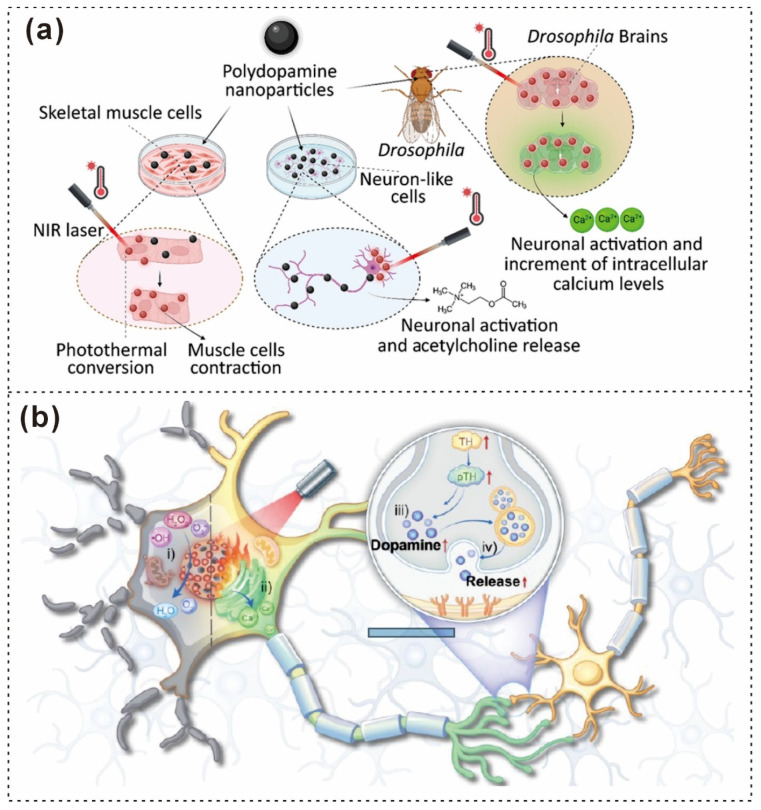
PDA applications in photothermal therapy. (**a**) Schematic illustration of how near-infrared-irradiated PDA nanoparticles modulate cellular activity on the basis of in vitro and ex vivo studies [198]. Copyright 2025, American Chemical Society. (**b**) Synergistic mitigation of oxidative damage and deep brain stimulation with photothermal activation for alleviating Parkinsonian symptoms [211]. Copyright 2025, American Chemical Society.

Carmingnani and colleagues highlighted that precise control of cell activity is essential for understanding and treating various disorders [198]. Recent developments in nanotechnology, particularly those focused on neurons and myotubes, have introduced photoresponsive nanoparticles as a noninvasive means to modulate cell functions with high spatial and temporal accuracy. This method reduces tissue damage and enhances the specificity of activation. They proposed an approach using fully organic PDA nanoparticles (PDNPs) to remotely control the activity of differentiated SH-SY5Y neurons and C_2_C1_2_ myotubes through near-infrared (NIR) laser stimulation. Confocal microscopy confirmed that individual neuron-like cells can be thermally activated, resulting in significant cellular responses such as calcium transients and acetylcholine release, as shown in Figure 21a [198].

Furthermore, deep brain stimulation (DBS) effectively reduces motor symptoms in Parkinson’s disease (PD) patients; however, it requires permanent invasive hardware implantation, and its effectiveness decreases as PD advances. Zhou et al. developed an implant-free NIR-II laser-activated nanosystem that combines wireless DBS with antioxidative neuroprotection to address these issues [211]. This system integrates enzyme-like 2-(phenylselanyl) ethan-1-amine (SePh), manganese dioxide (MnO_2_), and the NIR-II absorber IR-1048 (IR) onto a mesoporous polydopamine (mPDA) core, creating mPDA-SeMn-IR. When irradiated with an NIR-II laser, intraventricularly injected mPDA-SeMn-IR nanoparticles efficiently activate endogenous inositol 1,4,5-trisphosphate receptors, resulting in Ca^2+^ release from the endoplasmic reticulum (Figure 21b) [211]. When exposed to NIR irradiation, PDA produces heat that causes localized hyperthermia, damaging tumor cells and triggering apoptosis. Moreover, the biodegradability of PDA and its ability to be surface modified to enhance cellular uptake highlight its suitability for PTT applications, positioning it as a promising option for personalized, minimally invasive cancer treatment.

### 5.7. Tissue Repair

Owing to its high biocompatibility, adjustable features, and ability to create strong adhesive bonds with various surfaces, PDA has been employed to enhance the healing of damaged tissue. Studies have shown that it supports cell attachment, growth, and differentiation—key steps in the tissue regeneration process.

**Figure 22:**
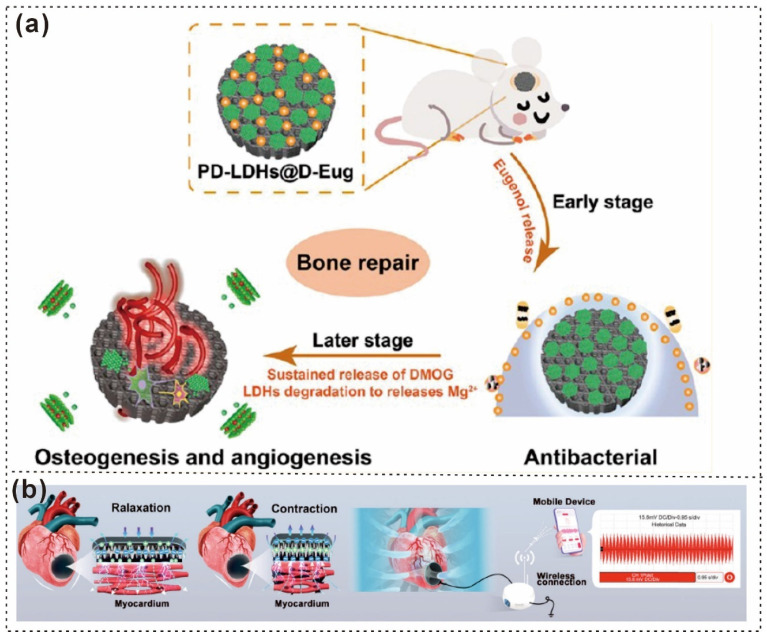
PDA applications in tissue repair. (**a**) Schematic illustration of a multifunctional 3D-printed scaffold designed for bone regeneration, featuring the spatiotemporal release of multiple drugs [137]. Copyright 2025, American Chemical Society. (**b**) An electrical system that captures the open-circuit voltage (VOC) generated via a schematic illustration of a versatile 3D-printed scaffold with spatiotemporal release of multiple drugs for bone regeneration. An art activity is conducted between the rGO electrode and the ground, and then the data are transmitted wirelessly to a smartphone [136]. Copyright 2024, Nature Portfolio.

Implanting a 3D-printed scaffold is one of the most effective approaches for treating bone defects. Bone repair is a highly complex process that requires a scaffold to support it, provides early antibacterial protection after implantation, and enhances later-stage angiogenesis and osteogenesis. Zelin Zhu and colleagues used layered double hydroxides (LDHs), a type of 2D inorganic nanomaterial, to efficiently load osteogenic and angiogenic dimethyloxalylglycine (DMOG) through anion exchange [137]. Additionally, DMOG-loaded LDH and eugenol, which are natural antibacterial agents, were co-modified onto the surface of 3D-printed poly(L-lactide) (PLLA) scaffolds via a PDA layer, creating a scaffold capable of spatiotemporally controlled release of different bioactive agents. Specifically, eugenol is released quickly in the early stage for antibacterial effects, whereas DMOG is released gradually from the LDHs to support long-term osteogenesis and angiogenesis. Moreover, the surface-anchored DMOG-loaded LDHs not only mechanically reinforced the PLLA scaffold but also enhanced osteogenic activity because Mg^2+^ was released during LDH decomposition. Notably, eugenol, DMOG, and LDH synergistically promote cell proliferation, angiogenesis, and osteogenic differentiation in vitro and accelerate vascularized bone formation in vivo (Figure 22a) [137].

Similarly, conductive cardiac patches can restore the electroactive environment in infarcted heart tissue; however, their repair efficacy depends on the incorporation of seed cells or drugs. The key to success lies in effectively combining electrical stimulation with the microenvironment fostered by these patches. Additionally, given the high readmission rate of heart patients, remote medical devices support successful recovery. Qiu and colleagues described a compact, self-powered biomimetic triboelectric nanogenerator featuring a unique double-spacer structure that integrates energy harvesting, therapy, and diagnosis within a single cardiac patch (Figure 22b) [136]. Moreover, PDA-based scaffolds and hydrogels demonstrate promise in delivering growth factors and drugs by providing a controlled-release system that speeds up tissue regeneration. The adaptable properties of PDA allow it to be functionalized with bioactive molecules, making it suitable for diverse tissue repair approaches, ranging from bone and cartilage to nerve and skin regeneration.

## 6. Potential Future Applications

This paper highlights the growing interest in PDA-based systems for potential future applications, although many are still in the preclinical or early clinical trial stages. These systems are being explored for drug delivery, wound healing, tissue engineering, and cancer therapy, with promising results in improving biocompatibility, adhesion, and functionalization. While there are no widely commercialized PDA-based products yet, several application trials are underway to test the efficacy of PDA composites in localized drug delivery and targeted therapy. The versatility of PDA materials, including their ability to modify surface properties and interact with a wide range of biomolecules, makes them strong candidates for future use. However, challenges such as scalability, long-term stability, and regulatory approval must be addressed before widespread commercialization.

When approving polydopamine-based (PDA) biomaterials by the FDA, the regulatory route is dependent on the intended purpose of the product. In case the material is similar to the available devices, then it can be cleared by premarket notification (510(k)) and proving substantial equivalence. Nonetheless, in new applications, high-risk applications, or novel uses, premarket approval (PMA) or an Investigational New Drug (IND) application is possible, with large preclinical and clinical data sets. Clinical trials are conducted in a stepwise process where Phase 1 is conducted to test safety, Phase 2 is conducted to test efficacy, and Phase 3 is conducted to test efficacy on a large scale. Phase 4 is conducted to monitor the use of the drug after it is on the market. Good Manufacturing Practices (GMP) should be followed, and it guarantees the quality, safety, and consistency in the production with validation of manufacturing procedures, sterility, and quality assurance. These steps will make sure that PDA biomaterials comply with the FDA requirements of both safety and performance in clinical practice.

## 7. Outlook and Future Perspectives

This article provides a comprehensive overview of PDA-based biomaterials, including their morphological classifications and synthesis methods, as well as their applications and the role of PDA in these applications. Researchers have investigated the mechanisms of PDA-drug conjugates at both the cellular and subcellular levels within the context of medical and research institutions. Polymers, such as PDA, are increasingly incorporated into drug delivery systems because of their ability to prolong the release of medication. Given the increasing interest in PDA-based biomedicines, this review highlights their applications in various settings because of their diverse morphological shapes.

Macromaterials focus primarily on wound healing, tissue regeneration, and adhesion enhancement. Their applications tend to be more focused on biomedical treatments, particularly in skin regeneration and tissue engineering. Similarly, nanoscale materials are more multifunctional and focus on drug delivery, imaging, and tumor targeting. These materials are engineered explicitly for therapeutic applications, including cancer treatment, photothermal therapy, and controlled drug release.

PDA-based macromaterials and nanoscale materials have advantages and disadvantages. The benefits of macromaterials include strong biological integration and the ability to aid in regeneration and self-healing. However, the advantages of nanoscale materials include targeted drug delivery, high stability, and specific biological targeting. Similarly, macromaterials tend to face structural instability, cytotoxicity issues, and complex preparation processes, and nanoscale materials face toxicity risks, issues with size control, and stability concerns.

Future research could target overcoming the stability challenges of PDA-based nanoscale and macroscale materials to ensure their durability across different environments. One promising direction is improving the chemical and physical robustness of PDA derivatives, especially at various temperatures, pH values, and mechanical stresses. Additionally, the potential toxicity of PDA-based materials, particularly in biological settings, should be carefully evaluated and reduced through molecular modifications or biocompatible coatings. The mechanical properties, such as strength, flexibility, and elasticity, of PDA hydrogels can also be tailored to suit specific applications, enhancing their performance in areas such as drug delivery and tissue engineering. Progress in these areas could also enable the creation of hybrid materials that combine PDA with other bioactive or synthetic substances for added functionality. Overall, these developments support the wider use of PDA-based materials in biomedical and industrial applications.

## Data Availability

No new data were created or analyzed in this study.

## Abbreviations

polydopamine-polypyrrole (PDA-PPy), Astragaloside IV (AST), F127CHO-GelDA-PDA-PPy@AST (FGPP@AST), mussel-inspired fullerene nanocomposites (C60@PDA), surface-oxidized arsenene nanosheets (As/As_x_O_y_ NSs), PDA-PEDOT functionalized sulfonic MXene nanosheets (MxNSPP), MXene nanosheets (MxNS), poly (vinylidene fluoridetrifluoroethylene) nanofiber (PVFT NF), poly(l-lactic acid)–poly(citrate siloxane)–curcumin@polydopamine (PPCP), magneto-mechano-electric (MME) cascade stimulation system based on aligned magnetoelectric nanofibrous membranes (PLLA/CFO), BiF_3_@PDA@PEG (BPP), Ultrasound imaging contrast agents (UCAs), quercetin-encapsulated (Que) and polydopamine-integrated (PDA@QLipo), poly(fluorene-*co*-vinylene) (PFV), manganese dioxide (MnO_2_)(MD), yeast cell wall (YCW) encapsulate MD@MPDA (YMD@MPDA), DA nanoscale coordination polymers (DA-NCPs), cerium oxide (Ce_x_O_y_), prussian blue (PB), 1,3,5-trimethylbenzene (TMB), hydrophilic metal–organic framework (UIO-66-NH_2_), supramolecular template (PBDT-TA), HPDA-ABS/PEG-BEZ235/Ce6 (H-APBC), (K_0.5_Na_0.5_NbO_3_, KNN), poly(lactic acid) (PLA), quaternized chitosan (QCS), oxidized dextran (OD), tobramycin (TOB).
